# A new subclass of intrinsic aminoglycoside nucleotidyltransferases, ANT(3")-II, is horizontally transferred among *Acinetobacter* spp. by homologous recombination

**DOI:** 10.1371/journal.pgen.1006602

**Published:** 2017-02-02

**Authors:** Gang Zhang, Sébastien Olivier Leclercq, Jingjing Tian, Chao Wang, Koji Yahara, Guomin Ai, Shuangjiang Liu, Jie Feng

**Affiliations:** 1 State Key Laboratory of Microbial Resources, Institute of Microbiology, Chinese Academy of Sciences, Beijing, China; 2 Department of Bacteriology II, National Institute of Infectious Diseases, Musashimurayama, Tokyo, Japan; Uppsala University, SWEDEN

## Abstract

The emergence and spread of antibiotic resistance among *Acinetobacter* spp. have been investigated extensively. Most studies focused on the multiple antibiotic resistance genes located on plasmids or genomic resistance islands. On the other hand, the mechanisms controlling intrinsic resistance are still not well understood. In this study, we identified the novel subclass of aminoglycoside nucleotidyltransferase ANT(3")-II in *Acinetobacter* spp., which comprised numerous variants distributed among three main clades. All members of this subclass can inactivate streptomycin and spectinomycin. The three *ant(3")-II* genes, encoding for the three ANT(3")-II clades, are widely distributed in the genus *Acinetobacter* and always located in the same conserved genomic region. According to their prevalence, these genes are intrinsic in *Acinetobacter baumannii*, *Acinetobacter pittii*, and *Acinetobacter gyllenbergii*. We also demonstrated that the *ant(3")-II* genes are located in a homologous recombination hotspot and were recurrently transferred among *Acinetobacter* species. In conclusion, our findings demonstrated a novel mechanism of natural resistance in *Acinetobacter* spp., identified a novel subclass of aminoglycoside nucleotidyltransferase and provided new insight into the evolutionary history of intrinsic resistance genes.

## Author summary

The level of interest in intrinsic resistance genes has increased recently, and one of reasons is that their mobilization could lead to emergence of resistant pathogens. Insertion sequences (ISs) or plasmids can capture intrinsic resistance genes and disseminate them in bacterial populations. In this study, we identified a novel subclass of aminoglycoside nucleotidyltransferases which are intrinsic in *A*. *baumannii* and other *Acinetobacter* species. The genes encoding the aminoglycoside nucleotidyltransferase were frequently horizontally transferred between different *Acinetobacter* species by homologous recombination. This work reports a novel mechanism of natural resistance in *Acinetobacter* and an overlooked pathway for the dissemination of resistance among species in this genus.

## Introduction

The genus *Acinetobacter* includes a broad group of biochemically and physiologically versatile bacteria that are ubiquitous in many environments [1]. *Acinetobacter baumannii* is the most clinically significant *Acinetobacter* species, and is among the most concerning causes of hospital-acquired bacterial infections [2]. Several other species of the genus may also cause human infections, such as *A*. *nosocomialis* and *A*. *pittii*, and less frequently *A*. *ursingii*, *A*. *haemolyticus*, *A*. *lwoffii*, *A*. *parvus*, and *A*. *junii* [3]. In the last few decades, the rate of hospital-acquired infections caused by *Acinetobacter* spp. have increased markedly worldwide [4, 5]. Of more concern is the rapidly increasing rate of multidrug resistance among *Acinetobacter* isolates, which has reduced the number of effective therapeutic options [1]. A study in US hospitals demonstrated that the rate of multidrug-resistant (MDR) *Acinetobacter* isolates increased from 7 to 30% between 1993 and 2004 [6]. In Chinese hospitals, more than half of *A*. *baumannii* isolates in 2014 were resistant to at least 10 frequently used antibiotics, and the rate of resistance to imipenem and meropenem has increased markedly (from 31–39% in 2005 to 62.4–66.7% in 2014) [7]. Additionally, the frequency of detection of multi-resistant non-*baumannii* isolates such as *A*. *pittii*, *A*. *junii*, *and A*. *johnsonii* has increased considerably, which demonstrated the exceptional adaptation of *Acinetobacter* spp. to antibiotic pressure.

The emergence and spread of antibiotic resistance among *Acinetobacter* spp. have been investigated extensively. Many antibiotic resistance genes (ARGs) are located on plasmids or genomic resistance islands in *Acinetobacter* isolates [8, 9]. In addition to ARGs located on mobile elements, several chromosomally encoded efflux systems and β-lactamases have been reported to contribute to the resistance of *A*. *baumannii*. The corresponding genes confer resistance when their expression changes due to mobilization or mutation of their associated regulatory elements. For instance, the *adeABC* operon, which encodes an efflux pump, is not expressed in environmental *A*. *baumannii* isolates; and the MDR phenotype is caused by mutations in *adeRS* located upstream of *adeABC*, leading to over-expression of the pump [10]. By providing a strong promoter, insertion of IS*Aba1* upstream of *bla*_OXA-51_ in *A*. *baumannii* also increased the expression of the β-lactamase gene, which conferred resistance to carbapenems [11, 12]. Interestingly, IS*Aba1* was also responsible for *bla*_OXA-51_ mobilization, as indicated by detection of a plasmid-borne IS*Aba1*-*bla*_OXA-51_-like gene in *baumannii* and non-*baumannii* species of *Acinetobacter* [13, 14]. Another example is the emergence of the mobile *bla*_NDM-1_ carbapenemase gene, which was initially captured by the genome of an *Acinetobacter* species where it was integrated in the transposon *Tn*125, enabling its efficient spread in various pathogens [15]. Finally, recent works have shown that the *aphA6* and *aac(6')-Ih* genes, which encode aminoglycoside-modifying enzymes, are intrinsic in *A*. *guillouiae* and *A*. *gyllenbergii*, respectively, but were acquired by *A*. *baumannii* by means of IS mobile elements [16, 17]. When captured by mobile elements, chromosomally-encoded conserved genes can thus act as a source of acquired resistance. Despite the important role of intrinsic genes in resistance, a substantial gap remains in our understanding of the intrinsic mechanisms responsible for the antibiotic resistance of *Acinetobacter* spp. Good candidates for intrinsic resistance are transferases, which are present in bacterial chromosomes at a high frequency, and which have a diverse range of antibiotic substrates. For example, nucleotidyltransferases, phosphotransferases, and acetyltransferases can modify aminoglycosides, macrolides, chloramphenicols, and lincosamides [18].

In the present study, we scanned the publicly available bacterial genomes of *Acinetobacter* spp. to detect putative antibiotic transferases. Putative aminoglycoside nucleotidyltransferase (*ant*) genes were found in conserved chromosomal regions in various *Acinetobacter* spp. Functional analyses indicated that the putative encoded transferases from different species can increase streptomycin and spectinomycin resistance of *Escherichia coli*. According to the distant phylogenetic relationship between these proteins and the reported ANT(3")-I nucleotidyltransferases, we propose that the *Acinetobacter* ANT(3") should refer to a new subclass of aminoglycoside nucleotidyltransferases, designated ANT(3")-II. The genes encoding for ANT(3")-II are intrinsic in at least *Acinetobacter baumannii*, *Acinetobacter pittii* and *Acinetobacter gyllenbergii*. Moreover, we also demonstrated that these intrinsic genes recurrently transferred among *Acinetobacter* species by homologous recombination.

## Results

### Putative aminoglycoside nucleotidyltransferases are widely distributed in the genus *Acinetobacter*

Comparison of the amino acid sequences of known aminoglycoside, macrolide, chloramphenicol, and lincosamide transferases with complete and draft genomes of *Acinetobacter* spp. from GenBank resulted in identification of 90 protein sequences with more than 30% amino acid identity with known resistance genes and less than 80% amino acid identity with each other. Among them, five were encoded by genes located in conserved chromosomal regions and had not hitherto been reported as antibiotic resistance determinants. These putative proteins shared the highest amino acid sequence identity (39–44%) with aminoglycoside nucleotidyltransferases/adenylytransferases (ANT) of the class ANT(3")-I (sometimes designated Aad), which catalyze adenylylation at the 3'' [ANT(3'')] position [19] (S1 Table). They are present in *A*. *baumannii*, *A*. *pittii*, *A*. *junii*, *A*. *parvus*, *A*. *gyllenbergii*, and *A*. *ursingii*, as well as 24 *Acinetobacter* isolates that have not been identified to the species level.

**Fig 1:**
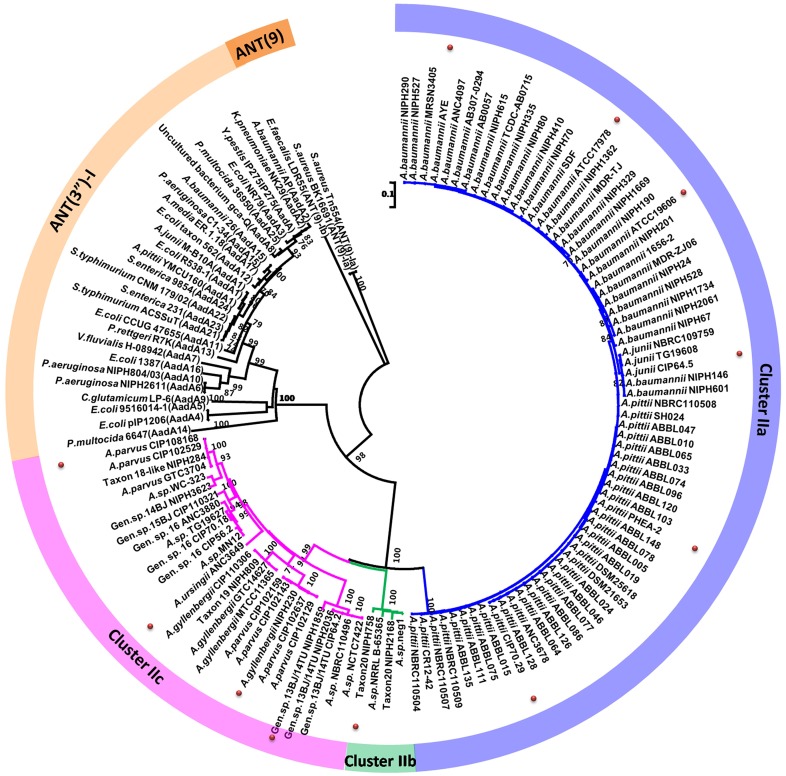
Phylogenetic neighbor-joining tree of aminoglycoside nucleotidyltransferases (ANT(3")). Numbers above each node are the percentages of tree configurations that occurred during 1000 bootstrap replicates. Only values greater than 70% are shown. The abbreviation of bacteria denoted as follow: *E*. *coli*, *Escherichia coli; P*. *multocida*, *Pasteurella multocida; P*. *aeruginosa*, *Pseudomonas aeruginosa; C*. *glutamicum*, *Corynebacterium glutamicum; V*. *fluvialis*, *Vibrio fluvialis; P*. *rettgeri*, *Providencia rettgeri; S*. *enterica*, *Salmonella enterica; A*. *media*, *Aeromonas media; Y*. *pestis*, *Yersinia pestis; K*. *pneumoniae*, *Klebsiella pneumoniae; E*. *faecalis*, *Enterococcus faecalis; S*. *aureus*, *Staphylococcus aureus*. Red dots represent the ANT(3")-II from *Acinetobacter* species expressed in *E*. *coli*.

We generated neighbor-joining, maximum-parsimony, and maximum-likelihood phylogenetic trees to evaluate the relationships between the putative ANTs and all known reported ANT(3")-I enzymes (Fig 1, S1 Fig). We used ANT(9) sequences from *Staphylococcus aureus* and *Enterococcus faecalis* as outgroups, which catalyze adenylylation at the 9[ANT(9)] position [19]. The three phylogenetic methods yielded trees with similar topologies, in which all protein sequences of the genus *Acinetobacter* clustered apart from known ANT(3")-I enzymes, as a highly supported monophyletic clade (with 100% bootstrap confidence), designated ANT(3")-II. These proteins share 64–72% amino acid identity with each other and 35–44% amino acid identity with known ANT(3")-Ia enzymes. ANT(3")-II formed three highly supported monophyletic clades, which were designated Cluster IIa, IIb, and IIc (Fig 1). Cluster IIa included putative ANTs of *A*. *baumannii*, *A*. *pittii*, and *A*. *junii*. All IIa members share at least 96% amino acid identities with each other. Cluster IIb consisted of only four sequences, all of which were from unknown *Acinetobacter* species, and shared more than 96% amino acid identities with each other. Finally, Cluster IIc included all remaining ANT(3")-II homolog sequences detected in *Acinetobacter* genomes, and shared 68–100% amino acid identities with each other. These three groups shared at most 72% amino acid identity with each other and their phylogenetic relationships was not consistent depending on the reconstruction method used (S1 Fig), supporting the clustering in three independent groups.

### Putative aminoglycoside nucleotidyltransferases provide streptomycin and spectinomycin resistance when expressed in *E*. *coli* and in their natural host

The putative *ant(3")-II* genes from *A*. *baumannii*, *A*. *junii*, and *A*. *pittii* (IIa); *Acinetobacter* sp. NIPH 758 (IIb); and *A*. *parvus*, *A*. *gyllenbergii*, and *A*. *ursingii* (IIc) were functionally expressed in *E*. *coli* (Fig 1). All of the above candidate genes increased the resistance of *E*. *coli* to streptomycin and spectinomycin by 32–128-fold, compared to the control (*E*. *coli* harboring the null vector pUC18 only) (Table 1). However, the expressed ANTs did not result in increased resistance to other aminoglycosides, suggesting a substrate specificity for the transferase, consistent with other enzymes of the ANT(3") class [19].

**Table 1:** Functional analysis of putative ANT(3")-II from *Acinetobacter* expressed in *E*. *coli*.

Original strains	Protein ID	Expression host	MIC (μg/ml)	
			streptomycin	spectinomycin
		*E*. *coli* DH5α	4	32
*A*. *baumannii* ATCC 19606	EEX02086	*E*. *coli* DH5α	512	2048
*A*. *baumannii* AYE	CAJ77832	*E*. *coli* DH5α	512	1024
*A*. *baumannii* ATCC 17978	ABO10616	*E*. *coli* DH5α	512	1024
*A*. *junii* CIP 64.5	ENV65326	*E*. *coli* DH5α	512	2048
*A*. *pittii* CIP 70.29	ENW14756	*E*. *coli* DH5α	512	2048
*A*. *pittii* PHEA-2	ADY83642	*E*. *coli* DH5α	256	2048
*A*. *parvus* CIP 108168	ENU37733	*E*. *coli* DH5α	256	2048
*A*. *ursingii* ANC 3649	ENV80847	*E*. *coli* DH5α	128	1024
*A*. *gyllenbergii* NIPH 230	ESK39014	*E*. *coli* DH5α	256	2048
Gen. sp. 13BJ/14TU NIPH1859	ENX32354	*E*. *coli* DH5α	256	2048
Taxon 20 NIPH 758	ENU91137	*E*. *coli* DH5α	256	2048

To assess the contribution of ANT(3")-IIa to intrinsic resistance in *A*. *baumannii*, the *ant(3")-IIa* gene was inactivated in *A*. *baumannii* ATCC 19606 and the susceptibility of the mutant strain to aminoglycosides was evaluated (Table 2). Inactivation of *ant(3")-IIa* decreased streptomycin and spectinomycin resistance by 16-fold and 2-fold, respectively, which can be fully restored by a complementation plasmid expressing *ant(3")-IIa* (Table 2), demonstrating that ANT(3")-IIa contributes to intrinsic resistance in *A*. *baumannii*.

**Table 2:** Resistance levels of *ant(3")-IIa* deficient mutant and complementation assay of the mutant.

Strains	MIC (μg/ml)					
	Km	Sm	Spe	Gm	Ak	Tb
*A*. *baumannii* ATCC 19606 (wild-type)	16	1024	5120	32	32	8
*A*. *baumannii* ATCC 19606 (△*ant*)	16	64	2560	32	32	8
△*ant*/pKFAb::*ant(3")-IIa*	-	>1024	>5120	-	-	-

Km, kanamycin; Sm, streptomycin; Spe, Spectinomycin; Gm, gentamycin; Ak, amikacin; Tb, tobramycin. △*ant*, *ant(3")-IIa* deficient mutant of wild-type *A*. *baumannii* ATCC 19606; △*ant*/pKFAb::*ant(3")-IIa*, complementation plasmid pKFAb::*ant(3")-IIa* expressed in △*ant*.

### ANT(3")-IIa is a valid streptomycin nucleotidyltransferase

To characterize the products of enzymatic inactivation, ANT(3")-IIa from *A*. *baumannii* ATCC 19606 was over-expressed in *E*. *coli* BL21 (DE3) and purified. The molecular weight of ANT(3")-IIa was 32 kDa (S2 Fig). Enzyme activity was assayed using liquid chromatography-mass spectrometry (LC-MS). With streptomycin [m/z 582.4 (M+H)^+^] as a substrate, a reaction product with an (M+H)^+^ ion at m/z 911.3 was detected in the reaction system (S3 Fig), indicative of addition of a single adenyl group to the antibiotic, which was confirmed by the product ion at m/z 911.3. This result suggests that the ANT(3")-IIa enzyme of *A*. *baumannii* can adenylate streptomycin, and validates its functional classification as a streptomycin nucleotidyltransferase.

### *ant(3")-IIa* and *ant(3")-IIc* are intrinsic in *A*. *baumannii*, *A*. *pittii*, and *A*. *gyllenbergii*

The *ant(3")-IIa* gene in *A*. *baumannii* isolates was located at the same chromosomal locus, within a more than 20-kbp-long region highly conserved among most strains (S4 Fig), and with no evidence of mobile element in the vicinity of the gene. Moreover, the gene was present in 1027 of 1406 *A*. *baumannii* genomes. The conserved location and high prevalence of the gene among *A*. *baumannii* isolates (73%) suggest it to be intrinsic in this species. Similarly, *ant(3")-IIa* in *A*. *pittii*, and *ant(3")-IIc* in *A*. *gyllenbergii* are located in conserved chromosomal regions and are also present in most sequenced isolates, again indicating that they are intrinsic in the corresponding species. Although the *ant(3")-II* genes are also located in the same chromosomal region in all other *Acinetobacter* genome sequences we investigated, determination of whether these genes are intrinsic is hampered by the impossibility to cluster the sequenced isolates into well defined species.

### The intrinsic *ant(3")-IIa* gene of *A*. *baumannii* was horizontally transferred to *A*. *junii*

**Fig 2:**
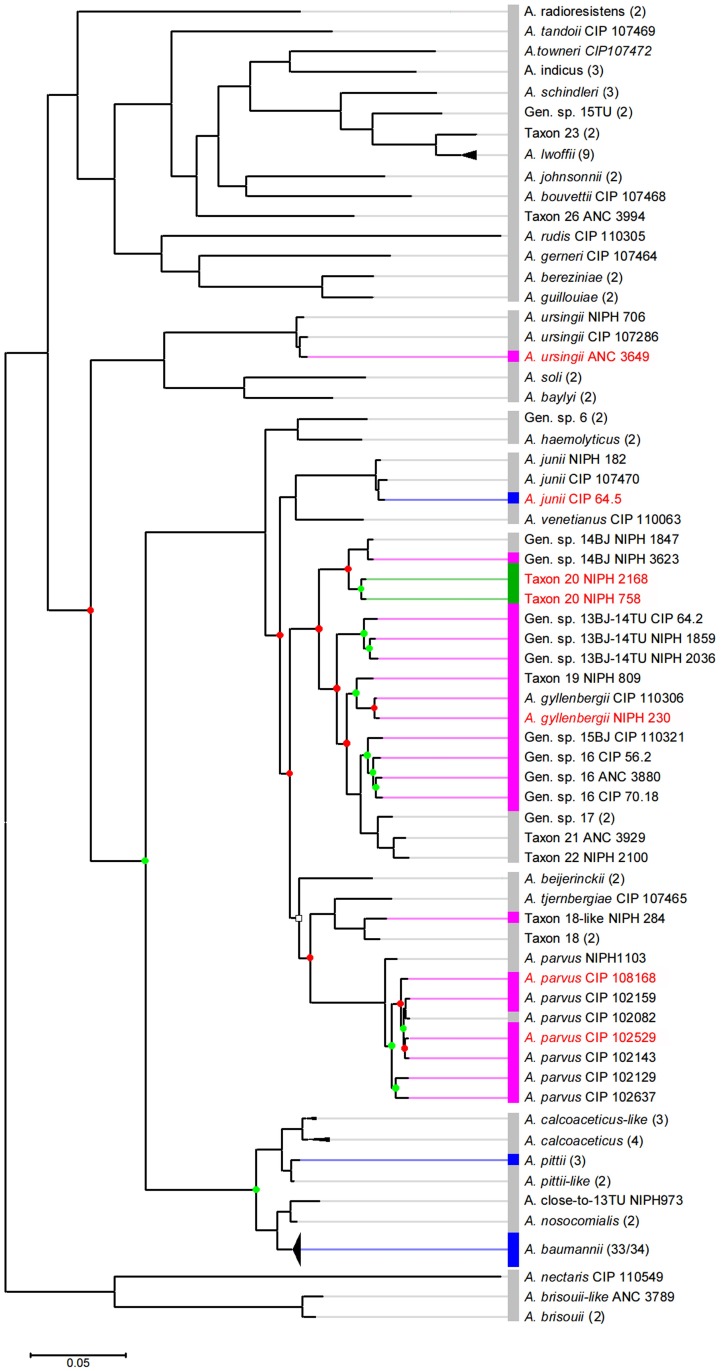
Phylogeny of the *Acinetobacter* genus based on the alignment of the core-genome proteins. Modified from Touchon et al. with permission [3]. The white squared node have a 67% bootstrap value; all other nodes show >95% bootstrap values. Green and red nodes are consistent and inconsistent with the ANT(3")-II phylogenetic tree of Fig 1, respectively. The *ant(3")-II* gene present in each taxon is indicated by a colored box: blue, *ant(3")-IIa*; green, *ant(3")-IIb*; pink, *ant(3")-IIc*; grey, no gene. Taxa written in red are those discussed in the text. Numbers in parenthesis indicate the number of genomes concatenated under the same taxon; they are all consistent in term of *ant(3")-II* variant, except in *A*. *baumannii* where the strain TYTH-1 lacks a 13.8 kbp region including *ant(3")-IIa*.

The unexpected inclusion of *A*. *junii* ANT(3")-IIa sequences among *A*. *baumannii* sequences in the phylogenetic tree (Fig 1) prompted further investigation of the phylogenetic relationships among ANT-carrying *Acinetobacter* strains. The position of strains carrying *ant(3")-II* genes was mapped on the *Acinetobacter* phylogenetic tree based on the whole core-genome proteins [3]. First, the observed pattern revealed that almost all strains carrying an *ant(3")-II* gene (with the exception of *A*. *ursignii* ANC3649) cluster in a single clade of the phylogeny (Fig 2). Second, numerous strains lacking an *ant(3")-II* gene are also scattered in this clade. Third, even when looking at branches including *ant(3")-II*-carrying strains only, the ANT(3")-II distribution of Fig 1 is globally inconsistent with the strains' phylogeny. Nearly half of the nodes linking *ant(3")-II*-carrying strains do not match the phylogenetic relationships observed for the ANT(3")-II sequences (red nodes in Fig 2). Such discrepancies between accessory and core gene phylogenies are generally indicative of horizontal gene transfer (HGT), usually mediated by mobile genetic elements. However, no mobile element or mobile element remnant could have been detected in the vicinity of the *ant(3")-II* genes in any of the genomes investigated.

**Fig 3:**
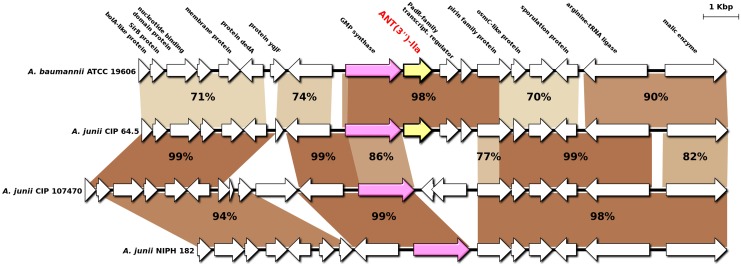
Genomic context and homologous recombination (HR) analysis of *ant(3")-IIa* in *A*. *baumannii* and *A*. *junii*. Deep brown indicates high nucleotide similarity, and light brown indicates low nucleotide similarity. Arrows indicate open reading frames and the direction of transcription. Yellow arrow, *ant(3")-IIa*; Pink arrow, GMP synthase gene. Boldfaced text above the arrows indicates the protein product of each gene. The displayed sequences of *A*. *baumannii* ATCC19606, *A*. *junii* CIP 64.5, CIP 107470 and NIPH 182 are subregions of Genbank accessions, JMRY01000011, APPX01000040, APPS01000002 and APPW01000012, respectively.

To explore whether HGT caused the observed inconsistencies even so, we first focused on the unexpected position of *A*. *junii* ANT(3")-IIa among *A*. *baumannii* ANT(3")-IIa proteins, considering that both species are distantly related in the core-genome tree (Fig 2). We aligned the 10-kbp downstream and upstream regions of *ant(3")-IIa* from the *A*. *baumannii* ATCC19606 genome, the *A*. *junii* CIP 64.5 genome, and two other representative *A*. *junii* genomes devoid of the gene (Fig 3). Consistent with the phylogenetic analysis, the 4.3-kbp region including *ant(3")-IIa* in *A*. *junii* CIP 64.5 was 98% identical to those of *A*. *baumannii*, while the flanking regions were only 70–77% identical. More interestingly, an inner portion of the 4.3-kbp region, which included *ant(3")-IIa* and the following two genes, was replaced by an unrelated 1.8-kbp DNA sequence in *A*. *junii* CIP 107470 and by a small, unrelated 300-bp sequence in *A*. *junii* NIPH 182 (Fig 3). Such pattern is typical of gene acquisition through homologous recombination (HR). To validate the occurrence of a HR event between *A*. *baumannii* and *A*. *junii* at the *ant(3")-IIa* locus, we performed an HR analysis on the 14-kbp region surrounding *ant(3")-IIa* by recombination detection program (RDP) analysis. Seven independent HR detection algorithms were selected, all of which provided highly significant support for a 4.3-kbp recombination event in *A*. *junii* CIP 64.5 with *A*. *baumannii* ATCC 19606 as the donor (S2 Table). All methods located the maximal recombined region between positions 103,874 and 108,115 bp in the *A*. *junii* CIP 64.5 sequence, which included *ant(3")-IIa*. These results confirm that *ant(3")-IIa* in *A*. *junii* CIP 64.5 originated from *A*. *baumannii* and was integrated in the genome through homologous recombination.

### The *ant(3")-II* genes are in a recombination hotspot in the genus *Acinetobacter*

Another clear-cut inconsistency between the ANT(3")-II and *Acinetobacter* phylogenies is the clustering of the *A*. *gyllenbergii* NIPH 230 ANT(3")-IIc with *A*. *parvus*-related ANT(3")-IIc. Analysis of the genomic context of *ant(3")-IIc* in *A*. *gyllenbergii* NIPH 230 and *A*. *parvus* CIP 102637 showed that the sequence of the gene and its surrounding region (1.5 kbp) is 100% identical between the two strains, while the remaining flanking regions are only 74–84% identical (S5 Fig). Other discrepancies include the presence of *ant(3")-IIc* in *A*. *ursingii* ANC 3649, the presence of the strains carrying *ant(3")-IIb* in an inner branch of the *ant(3")-IIc* clade, and the position of *A*. *parvus* CIP 108168 and CIP 102529 away from the other *A*. *parvus* strains in the ANT(3")-II phylogeny (Figs 1 and 2). All these discrepancies can be attributed to HGT mediated by HR, although the lack of representative genomes and sequence incompleteness prevented us from fully resolving the HR events leading to the observed patterns (S6–S8 Figs).

**Fig 4:**
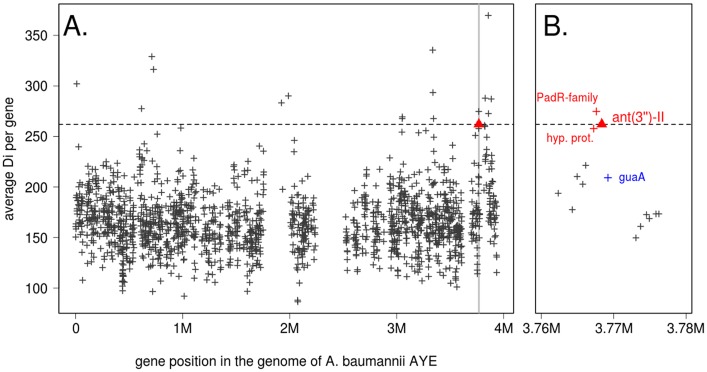
Intensity of homologous recombination (HR) along *Acinetobacter* genomes. The X axis indicates the position in the reference genome *A*. *baumanni* AYE, and the Y axis the gene-average value of the orderedPainting statistics *D*_*i*_, measuring the intensity of recombination at nucleotide *i*. The dotted line represents the top percentile value. The *ant(3")-II* locus is marked by a red triangle. (A) Recombination intensity along the whole genome. (B) HR intensity in the 20 kbp surrounding *ant(3")-II* shaded in grey in (A). Genes considered as in a recombination hotspot are marked in red, and *guaA* is marked in blue.

The high number of observed HR events involving *ant(3")-II genes* across *Acinetobacter* species prompted us to investigate the rate of recombination (precisely, the intensity of DNA transfer) at this locus compared to the global genome recombination rate. We first ran the ordered Painting algorithm [20] on all the *A*. *baumannii* genomes present in Fig 1 (see Methods). In *A*. *baumanni*, *ant(3")-IIa* ranked as the 126^th^ most recombined gene out of the 2282 genes present in all investigated genomes. Although such rank is indicative of a high recombination rate, it cannot be considered as a recombination hotspot according to the criterion proposed in [20]. When the analysis was performed on all *Acinetobacter* genomes carrying an *ant(3")-II* gene, the locus ranked 17^th^ most recombined gene out of the 1694 genes present in all genomes, and fell in the range of the predicted recombination hotspots (Fig 4A). Interestingly, the two genes directly adjacent to *ant(3")-IIa* in *A*. *baumannii* AYE (encoding a PadR-family transcriptional regulator and a small conserved hypothetical protein) ranked 12^th^ and 21^th^ most recombined genes, respectively (Fig 4B). On the contrary, *guaA* (encoding a GMP synthase) ranked 118^th^ despite being also adjacent to *ant(3")-II* genes (Fig 4B). These results indicate that the *ant(3")-II* genes are part of a small recombination hotspot of ca. 2 kbp involved in the frequent horizontal transfer of the resistance gene across *Acinetobacter* species.

## Discussion

Intrinsic resistance genes are defined as conserved chromosomal genes, conferring phenotypic resistance in a given species [21]. Traditional intrinsic resistance genes encode either multidrug resistance efflux pumps that pump out intracellular antibiotics or outer membrane proteins that reduce the permeability of the outer membrane, decreasing antibiotic uptake [22]. Recently, a large number of other types of intrinsic resistance genes have been identified in *E*. *coli*, *Pseudomonas aeruginosa*, and *Staphylococcus aureus* by screening libraries of mutants obtained through precise gene deletions or transposon-tagged insertions that belong to a variety of cellular functional categories [23–26]. In the present study, bioinformatics screening of complete genome sequences and extensive molecular biology and biochemistry experiments were used to identify a new subclass of aminoglycoside nucleotidyltransferases, ANT(3")-II, in *Acinetobacter* spp. ANT(3")-II enzymes are phylogenetically distant from all known ANT(3")-I and the genes encoding for ANT(3")-II are always located at the same chromosomal locus in *Acinetobacter* spp., while the genes of ANT(3")-I are part of mobile genetic elements widely distributed in gram-negative bacteria. Furthermore, our findings revealed that ANT(3")-II is intrinsic in *A*. *baumannii*, *A*. *pittii* and *A*. *gyllenbergii*, bringing to light a novel natural mechanism of resistance in these clinically important pathogens.

Understanding the origins of antibiotic resistance would enable tracing their evolution, and predicting the emergence of clinical resistance. It has been proposed that certain antibiotic resistance genes in human bacterial pathogens originated from antibiotic-producing microorganisms, which use these genes to prevent suicide during drug production [27, 28]. Recent studies have shown that intrinsic genes, particularly in the genus *Acinetobacter*, can become mobile. The intrinsic genes *aph(3’)-VI* and *aac(6’)-Ih*, which encode aminoglycoside-modifying enzymes that originated from *A*. *guillouiae* and *A*. *gyllenbergii*, respectively, were captured by an IS element and disseminated in other *Acinetobacter* species [16, 17]. Similarly, *A*. *radioresistens* might be the origin of the *bla*_OXA-23_ carbapenem resistance determinant in *A*. *baumannii* [29]. The findings of our study demonstrated that, contrary to the above examples, the *ant(3")-II* genes were not mobilized through mobile elements but are located in a recombination hotspot, leading to frequent allele replacement or *ant(3")-II* gene acquisition in various *Acinetobacter* species. The impact of HR on the diversification of surface molecules and on the transfer of two resistance genes (an activated cephalosporin resistance gene and the *bla*_NDM-1_ gene) have been reported previously in *A*. *baumannii* [30–32]. However, the role of this molecular process in antibiotic resistance dissemination between *Acinetobacter* species is shown here for the first time. Moreover, the results suggest that this dissemination can occur even between very distantly related species of the genus, as exemplified by the *ant(3")-IIc* acquisition in *A*. *ursingii*. This phenomenon parallels the extensive horizontal exchanges of an intrinsic macrolide resistance gene among environmental and pathogenic species of the *Bacillus cereus* group we reported previously [33]. In the *B*. *cereus* group, acquisition of the intrinsic resistance gene was also mediated by HR. The fact that this process occurs in both Gram-positive and Gram-negative bacteria, and in both environmental and clinical isolates, suggests that transfer of resistance via HR may be more common than previously appreciated.

However, the high divergence of *ant(3")-II* sequences and their distribution mostly among non-clinical *Acinetobacter* spp. indicate that the first acquisition of this gene in a subgroup of *Acinetobacter* and its high rate of horizontal transfer likely predates the development of antibiotic for therapeutic use. It has become clear that clinical antibiotic resistance genes originated from intrinsic genes of environmental bacterial species, for which their primary function in the natural environment have been questioned [18, 28]. These general considerations would also apply for the *ant(3")-II* genes. On the one hand, *ant(3")-II* may function as a streptomycin/spectinomycin resistance gene in nature, since these two antibiotics are naturally produced by soil-dwelling bacteria [34, 35]. The high rate of horizontal transfer would thus be an ecological response to the patchy distribution of antibiotic producers in the soil. Similarly, the sequence diversification of ANT(3")-II may have been an evolutionary response to the production of new antibiotics variants by the producer, following the arms race hypothesis [36]. Exchange of *ant(3")-II* variants or even creation of mosaic variants would thus help the *Acinetobacter* spp. to adapt to the antibiotic compounds produced locally. On the other hand, the *ant(3")-II* genes primary function in nature may be unrelated to antibiotics resistance. More and more examples are now reported in the literature, suggesting that it could be a general trend [37–39]. Under this hypothesis, the underlying reasons of the diversification and high rate of horizontal transfer of *ant(3")-II* genes remains the same, except that the adaptive pressure is not known. A last possibility could be that the selection pressure actually acts on the flanking region rather than on the *ant(3")-II* locus itself. Indeed, the recombination hotspot region expands over 1.5 kbp downstream of *ant(3")-II* genes and include two other genes. The closest one particularly (ABAYE3738 in *A*. *baumannii* AYE, encoding a PadR-family transcriptional regulator), show even higher recombination intensity than *ant(3")-II*. The high rate of *ant(3")-II* genes horizontal transfers could thus be just a by-product of its physical position before this gene.

HR facilitates only permanent integration of a gene from an extra chromosomal DNA fragment into the chromosome, but the mechanism that could mediates cellular uptake of DNA fragments in *Acinetobacer* spp. remains unclear. Natural transformation has been described in some strains of *A*. *baylyi* [40], *A*. *baumannii* [41, 3], and *A*. *calcoaceticus* [42], suggesting that DNA uptake could occur via transformation. Genomic analysis of the genus *Acinetobacter* indicated the presence of most of the 13 key T4P (type IV pili) and competence-associated components in all genomes [3]. These studies suggest that members of the genus *Acinetobacter* have the potential to develop natural competence. Alternatively, general transduction has been suggested between *A*. *baumannii* strains, facilitated by one of the prophages present in the chromosome [32]. The extent to which these two processes provide the raw DNA material for HR and contribute to the genome plasticity observed in this genus is of great interest and requires further investigation.

Intrinsic resistance genes could provide an attractive therapeutic target to encourage the development of novel drugs that rejuvenate the activity of existing antibiotics [43]. We have shown that the deletion of *ant(3")-II* significantly decreases the resistance level of *A*. *baumannii* to streptomycin. Although no inhibitor of aminoglycoside-modifying enzymes is currently available, there is extensive effort to find how to overcome the action of these enzymes in pathogens [44]. Therefore, the demonstration that *A*. *baumannii* become more susceptible to streptomycin when *ant(3")-IIa* is silenced blazes a trail for future treatments against this extremely multi-resistant pathogen.

This work identified a novel subclass of intrinsic aminoglycoside nucleotidyltransferases in environmental and clinically important *Acinetobacter* species. The corresponding genes *ant(3”)-II* not only conferred phenotypic resistance in a given species but were also frequently horizontally transferred between different *Acinetobacter* species by homologous recombination. Overall, these results provide insight of *Acinetobacer*’s natural resistance and enhance our understanding of the evolutionary history of intrinsic resistance determinants in bacteria.

## Materials and methods

### Bacterial strains and culture conditions

*Escherichia coli* DH5α was used as a host for the expression of putative transferase resistance genes *in vitro*. The genomic DNA of *A*. *baumannii* ATCC 19606 was extracted to use as template for polymerase chain reaction (PCR). All strains were grown on Luria-Bertani (LB) broth at 37°C. The MIC for each strain was determined using broth microdilution assays, as recommended by the Clinical and Laboratory Standards Institute (CLSI, http://www.clsi.org/; CLSI 2010).

### Construction of recombinant plasmids carrying putative transferase resistance genes

Eleven open reading frames, representative of the putative *ant(3")-II* subclass were chosen from various *Acinetobacter* to determine their antibiotic resistance phenotypes. One DNA sequence, corresponding to the putative *ant(3")-IIa* from *A*. *baumannii* ATCC 19606, was amplified using the primers *a*ntF/R listed in S3 Table. Other genes from *A*. *baumannii* ATCC 17978, *A*. *baumannii* AYE, *A*. *junii* CIP 64.5, *A*. *pittii* CIP 70.29, *A*. *pittii* PHEA-2, *A*. *parvus* CIP 108168, *A*. *ursingii* ANC 3649, *A*. *gyllenbergii* NIPH 230, *A*. sp. NIPH 1859, and *A*. sp. NIPH 758 were synthesized by Sangon Biotech (Shanghai, China). The PCR product and synthesized DNA were cloned into multiple cloning sites of the pUC18 vector, resulting in recombinant plasmids carrying the target genes. *E*. *coli* DH5α was transformed with these recombinant plasmids, and clones were selected by blue-white screening on LB agar plates supplemented with 100 μg/ml ampicillin. The transformants containing the inserted fragments were confirmed by enzyme digestion and sequencing. Resistance of transformants to aminoglycosides (streptomycin, spectinomycin, kanamycin, gentamycin, amikacin, and tobramycin) were determined using the microdilution method, with induction by 0.5 mM isopropyl-β-D-thiogalactopyranoside (IPTG), as recommended by the CLSI (http://www.clsi.org/; CLSI 2010).

### Construction of resistance gene-deficient mutants

The plasmid pEX18Gm was used as a suicide vector to knock out *ant(3'')-IIa* in *A*. *baumannii*. The upstream and downstream regions (~500 bp) of the gene were amplified using genomic DNA of *A*. *baumannii* ATCC 19606 as a template, and combined by overlap PCR. The desirable fusion PCR product was ligated to pEX18Gm, resulting in a recombinant plasmid. The plasmid was transformed into *E*. *coli* S17-1λpir, to create a donor strain. *A*. *baumannii* ATCC 19606 was used as a recipient strain. Using a conjugation experiment, a merodiploid (containing the plasmid integrated with one HR event) was obtained by plating cells on LB plates containing 100 μg/ml ampicillin and 20 μg/ml gentamycin. The merodiploid was inoculated sequentially into LB liquid medium free of antibiotic for propagation at 37°C. After several rounds of propagation, serial dilutions were spread on LB agar plates with 20% sucrose. After overnight incubation at 37°C, colonies were selected on gentamycin-containing LB agar plates and LB agar without antibiotics. The gentamycin-sensitive clones, which have lost pEX18Gm through a second HR event, were verified as correct *ant(3'')-IIa*-deficient mutants by PCR and sequencing. The primers used for construction of gene-deficient mutants are listed in S3 Table.

### Construction of complementation plasmid

A 1337-bp DNA fragment containing the *A*. *baumannii* origin of replication was synthesized by Sangon Biotech (Shanghai, China) and then cloned into plasmid pKF18k-2 (Takara) using *Pvu*II restriction site. Inserts were selected in kanamycin 50 μg/ml and verified with PCR and enzyme digestion. The resulting plasmid was designated pKFAb, which can replicate in *E*. *coli* and *A*. *baumannii*. To construct the complementation plasmid the *ant(3")-II* gene was amplified from the *A*. *baumannii* ATCC 19606 chromosome using antF/R primers (S3 Table) and cloned into plasmid pKFAb with *Eco*RI and *Hin*dIII restriction sites by transformation of *E*. *coli* Top10. The resulting recombinant plasmid pKFAb::*ant* was verified by enzyme digestion and sequencing. At last the construct pKFAb::*ant* was introduced into competent *A*. *baumannii* (Δ*ant*) by electroporation, and transformants were selected on LB plates supplemented with streptomycin 150 μg/ml.

### ANT(3")-IIa enzyme overexpression and purification

*ant(3")-IIa* from *A*. *baumannii* ATCC 19606 (EEX02086) was inserted into pET28a (+) expression plasmid. The resulting recombinant plasmid was transformed into *E*. *coli* BL21 (DE3). A fresh overnight culture was used to inoculate 500 ml LB media in a 1/100 inoculum size. Culture was incubated at 37°C with shaking at 200 rpm up to OD_600_ of 0.6–0.8, followed by continued cultivation for an additional 16 h by addition of 0.5 mM IPTG at 25°C. Subsequently, cells were harvested by centrifugation, washed with 50 mM Tris-HCl buffer (pH 7.5). Then cells were resuspended in 30 ml of 50 mM Tris-HCl buffer (pH 7.5) and disrupted by using a ultrasonic cell disruptor (SCIENTZ, Ningbo). The cell lysate was clarified by centrifugation at 12,000×g for 20 min, and the supernatant (soluble protein fraction) was used for protein purification. Recombinant protein was purified with the His-Bind protein purification kit (Novagen, Madison, WI, USA) according to the manufacturer's instructions. To remove any residual proteins, imidazole and salts from the collected fractions, these fractions were pooled and re-separated with Superdex 200 gel chromatography with Tris-HCl buffer using a fast protein liquid chromatography system (Äkta FPLC, Amersham Biosciences, USA). The purified protein was concentrated by ultrafiltration using a Millipore Ultra-15 (10 kDa) centrifugal filter (Millipore, USA) and stored at −70°C.

### Detection of reaction product by LC-MS

The ANT(3")-IIa enzymatic reaction consisted of 50 mM HEPES (pH7.5), 1 mM dithiothreitol, 15 mM MgCl_2_, 1 mg streptomycin, 3 mM ATP, 0.5 mg of purified ANT(3")-IIa protein, and 2 units of inorganic pyrophosphatase in a total volume of 1 ml [45]. The reaction was performed at 37°C for 24 h and stopped by the addition of cold acetonitrile. The supernanant fraction was then applied onto a Millipore Ultra15 (10 kDa) centrifugal filter tube. After centrifugation at 12,000×g for 20 min, the protein was removed. The streptomycin and reaction product was eluted with deionized water. Prior to analyses, samples were filtered through a 0.22 μm filter and then subjected to LC-MS analysis on an Agilent 1260/6460 Triple quadrupole LC/MS system. The analytic column was ZORBAX SB-Aq (Agilent, 2.1×100 mm, 3.5 μm). A mixture of 2% methanol and 0.1% formic acid (v/v) in water under an isocratic elution program was used as the mobile phase. The flow rate was set at 0.1 ml min^-1^, and the column temperature was set at 30°C. The fragmentor voltage was 260 V. A capillary voltage of 4.0 kV and atomizing gas pressure of 35 psi were used. The flow rate of drying gas was 12 ml min^-1^, and the temperature of the solvent removal was 350°C. N_2_ was used as the collision gas and a collision voltage of 45 V was used for product ion scan. The LC-MS data were acquired and analyzed using a MassHunter Workstation Software (Version B.04.00, Agilent, Santa Clara, CA, USA).

### Bioinformatic methods and phylogenetic analysis

To discover new antibiotic transferases, two databases were constructed. One database consisted of a collection of amino acid sequences of known antibiotic transferases, including aminoglycoside, macrolide, chloramphenicol, and lincosamides transferases obtained from the Antibiotic Resistance Genes Database (ARDB; http://ardb.cbcb.umd.edu/index.html), and completed with more recently described transferase resistance genes. The other was the complete collection of assembled and draft *Acinetobacter* genome sequences downloaded from the NCBI FTP Site (ftp://ftp.ncbi.nlm.nih.gov/) in summer 2014. The antibiotic transferase sequences were searched against the genomes using TBLASTN. And the proteins sharing more than 30% amino acid identity and 50% coverage with known antibiotic transferases were selected for expression analyses.

Putative ANT(3")-II amino-acid sequences from *Acinetobacter* species were extracted from the respective genomes and the full set of ANT(3'')-I (AadA) protein sequences described in [19] were downloaded from Genbank. The protein dataset was completed with the recently described AadA25 protein [46], four additional AadA sequences located in *Acinetobacter* species, and two ANT(9)-I sequences to serve as outgroups. The accession numbers for all these proteins are listed in S4 Table. The sequence dataset was aligned using the CLUSTALW software with standard parameters [47]. Phylogenetic trees were constructed using NJ, MP and ML methods implemented in MEGA 6 software [48]. All phylogenetic trees were constructed using the partial deletion parameter set to 80% and 1000 bootstrap replicates. Other parameters were set to default for NJ and MP analyses, while the WAG+F model, with gamma-distributed rates among sites, was used for ML analysis.

The construction of the *Acinetobacter* core-proteins phylogenetic tree was described previously [3]. Briefly, orthologous core proteins shared by the analyzed *Acinetobacter* genomes were retrieved from bi-directional best hits. The 950 resulting families of proteins were concatenated and aligned to construct an approximated maximum-likelihood using FastTree with the WAG matrix of protein evolution, a gamma correction for variable evolutionary rates, and 100 bootstraps.

### Detection of homologous recombination events

HR detection at the *ant(3")-IIa* locus was performed using the RDP4 package, which includes several HR detection methods [49], on a 14 kbp conserved region surrounding the gene in *A*. *baumannii* and *A*. *junii* genomes. The region was extracted from the four sequences presented in Fig 1 and aligned using CLUSTALW, with a gap penalty of 20. The alignment was then carefully corrected visually, as alignment errors might critically alter HR detections, as explained in the RDP4 manual. The alignment was examined using the RDP [50], GENECONV [51], BootScan [52, 53], Maximum Chi Square (MAXCHI) [54], Chimaera [55], SisterScan [56], and PhylPro [57] methods. We retained only events unambiguously detected by the seven methods, using the following RDP4 parameters: a corrected P-value < 0.000001, with phylogenetic evidence, and with overlapping signals disentangled. HR investigations were made by running automated RDP analysis of the alignment of four sequences, and significant recombination events were detected.

### Inference of landscape and hot regions of homologous recombination

First, complete and draft genome sequences of the 31 *A*. *baumannii* strains carrying *ant(3")-IIa* (Fig 1) were downloaded from the Genbank database. The genome sequence of *A*. *baumannii* AYE was selected as reference, and each of the remaining genomes were aligned on this reference using progressiveMauve v2.4.0 [58]. Resulting pairwise alignments were then combined, and all positions in AYE identical in all strains as well as positions with missing data (gaps in one or more sequence) were discarded. The SNPs and their position in the remaining core genomic regions were provided as input to orderedPainting [20]. Next, the same analysis was performed on the whole *Acinetobacter* genus, again with *A*. *baumannii* AYE as reference. Genome sequence of all *Acinetobacter* from Fig 1 carrying *ant(3")-II* were downloaded from Genbank, except that only three *A*. *baumannii* (AYE, ATCC19606, and NIPH601) and three *A*. *pittii* (PHEA-2, ABBL015, and NBRC110504) were retained. The output of orderedPainting is a recombination intensity at each polymorphic site measured as a statistic *D*_*i*_. We therefore averaged the intensity value for each gene annotated in AYE. The top one percentile of gene-based recombination intensity was selected as indicative of recombination hotspot, as proposed in Yahara et al.'s report [20].

## Supporting information

S1 Fig Phylogenetic maximum likelihood (a) and maximum parsimony (b) trees of the aminoglycoside nucleotidyltransferases. Numbers above each node are the percentages of tree configurations that occurred during 1000 bootstrap replicates. Only values greater than 70% are provided.
(TIF)

S2 Fig SDS-PAGE of ANT(3")-IIa protein purification from *A. baumannii* ATCC 19606. lane 1, molecular weight markers (10 kDa–200 kDa); lane 2, *E*. *coli* BL21 (DE3) harboring null vector pET28a; lane 3, extract of *E*. *coli* BL21 (DE3) harboring pET28a-*ant*; 4, Purified ANT(3")-IIa protein.
(TIF)

S3 Fig LC-MS analysis [m/z 911.3 (M+H)^+^] of products from the reaction of ANT(3")-IIa with streptomycin. (TIF)

S4 Fig Genomic context of *ant(3")-IIa* in representative *A. baumannii* strains. Aqua green indicates high nucleotide similarity (>98%). Arrows indicate open reading frames and the direction of transcription. Yellow arrow, *ant(3")-IIa*; Red arrow, GMP synthase gene. Boldfaced text above the arrows indicates the protein product of each gene. The displayed sequences of *A*. *baumannii* ATCC 19606, 6200, AB0057, AB307-0294, ANC 4097, 17978, AYE, MDR-TJ, NIPH 1669, NIPH 290, NIPH 329, NIPH 527, NIPH 67, TCDC-AB0715, MDR-ZJ06, NCGM 237, 1656–2 and NIPH 201 are subregions of Genbank accessions, JMRY01000011, NZ_CP010397, NC_011586, NC_011595, APRF01000002, CP000521, NC_010410, NC_017847, APOQ01000016, APRD01000022, APQY01000009, APQW01000014, APRA01000009, CP002522, NC_017171, NZ_AP013357, NC_017162 and APQV01000013, respectively.
(TIF)

S5 Fig Genomic context and homologous recombination (HR) analysis of *ant(3")-IIc* in *A. gyllenbergii* and *A. parvus*. Deep orange indicates high nucleotide similarity, and light orange indicates low nucleotide similarity. Arrows indicate open reading frames and the direction of transcription. Boldfaced text above the arrows indicates the protein product of each gene. Yellow arrow, *ant(3")-IIc*; pink arrow, GMP synthase gene; genes without label, hypothetical proteins. The displayed sequences of *A*. *gyllenbergii* CIP 110306, *A*. *gyllenbergii* NIPH 230, *A*. *parvus* CIP 102637, and *A*. *parvus* CIP 102129 are subregions of Genbank accessions ATGG01000028, AYEQ01000163, APPG01000010, and APPA01000001, respectively.
(TIF)

S6 Fig Genomic context and homologous recombination (HR) analysis of *ant(3")-IIb* variants. Blue indicates high nucleotide similarity, and light green indicates low nucleotide similarity. Arrows indicate open reading frames and the direction of transcription. Yellow arrow, *ant(3")-II* genes; Pink arrow, GMP synthase gene; genes without label, hypothetical proteins. The putative HR event encompassed *ant(3")-IIb* and the downstream inserted region in Taxon 20 NIPH 2168 and NIPH 758, leading to a low similarity region inside an otherwise more highly similar region, when compared to Gen. sp. 14BJ NIPH 3623. Sequences of *A*. sp. neg1 and B-65365 are essentially identical to sequences of Taxon 20 NIPH 2168 and NIPH 758 and are not represented here. The displayed sequences of Taxon 20 NIPH 2168, NIPH 758, and Gen. sp. 14BJ NIPH 3623 are subregions of Genbank accessions APRW01000008, APPC01000022, and APSA01000001, respectively.
(TIF)

S7 Fig Genomic context and homologous recombination (HR) analysis of *ant(3")-IIc* in *A. ursingii* and *A*. sp. strains. Blue indicates high nucleotide similarity, and light green indicates low nucleotide similarity. Arrows indicate open reading frames and the direction of transcription. Yellow arrow, *ant(3")-IIc*; Pink arrow, GMP synthetase gene; genes without label, hypothetical proteins. The putative HR region encompasses the *ant(3")-IIc* gene and the GMP synthase-encoding gene in *A*. *ursignii* ANC 3649. The displayed sequences of *A*. *ursingii* CIP 107286, ANC 3649, Gen. sp. 15BJ CIP 110321, and Gen. sp. 16 ANC 3880 are subregions of Genbank accessions APQA01000027, APQC01000003, AQFL01000027, and APSD01000005, respectively.
(TIF)

S8 Fig Genomic context and homologous recombination (HR) analysis of *ant(3")-IIc* in the *A. parvus* clade. The phylogenetic relationship between strains as reported in Fig 2 is shown on the left side. Deep Orange indicates high nucleotide similarity, and light orange indicates low nucleotide similarity. Arrows indicate open reading frames and the direction of transcription. Yellow arrow, *ant(3")-IIc*; pink arrow, GMP synthase gene; striped arrow, restriction-modification gene; genes without label, hypothetical proteins. *A*. *parvus* CIP 102637 is repeated at the bottom of the figure to show its similarity with *A*. *parvus* CIP 102143 at the *ant(3")-IIc* locus. The nucleotide pairwise similarity at the *ant(3")-IIc* locus and the directly adjacent genes (including the GMP synthase and the PadR-family transcriptional regulator) is consistently lower than at the flanking regions, indicating multiple events of allele exchange through homologous recombination. *ant(3")-IIc* have also been precisely replaced by an unrelated gene in *A*. *parvus* CIP 102082. The displayed sequences of A. sp. *A*. *parvus* CIP 102637, CIP 108168, CIP 102159, CIP 102082, CIP 102529, and CIP 102143 are subregions of Genbank accessions APPG01000010, APOM01000001, KB851223, KB849372, KB849355, and APSE01000019, respectively.
(TIF)

S1 Table Putative transferase resistance proteins located on *Acinetobacter* chromosomes. (DOCX)

S2 Table Computational analysis of different HR algorithms with RDP4. (DOCX)

S3 Table Primers used to amplify the putative *ant(3")-IIa* and to construct gene-deficient mutant and complementation plasmid. (DOCX)

S4 Table Sequence accession or GI number used in Fig 1. (DOCX)
