# Supplementary figures and images for: A new subclass of intrinsic aminoglycoside nucleotidyltransferases, ANT(3")-II, is horizontally transferred among Acinetobacter spp. by homologous recombination
Source: PLoS Genet. 2017 Feb 2;13(2):e1006602. doi: 10.1371/journal.pgen.1006602 (PMC5313234; doi:10.1371/journal.pgen.1006602)

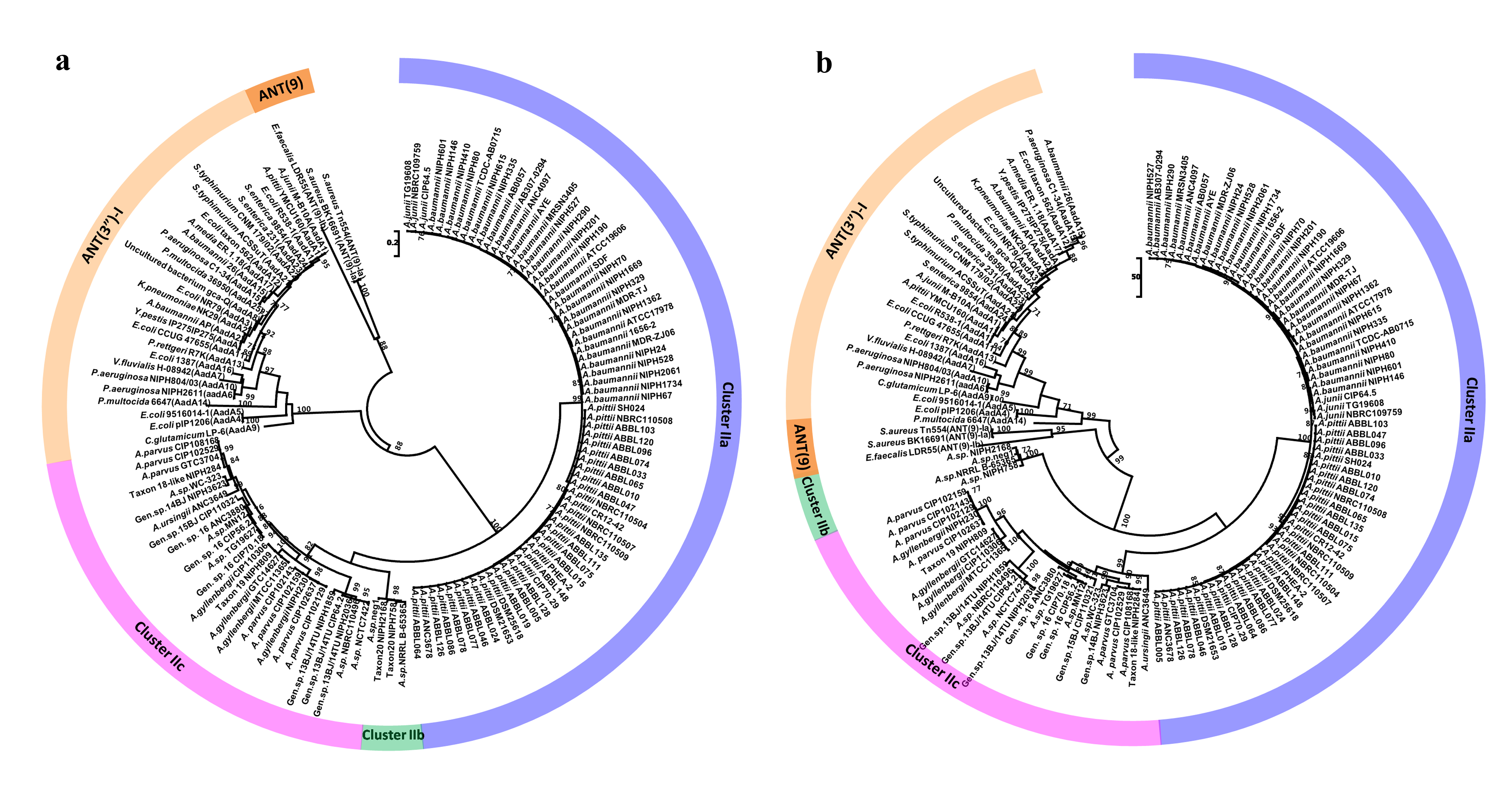

Supplement: S1 Fig — Numbers above each node are the percentages of tree configurations that occurred during 1000 bootstrap replicates. Only values greater than 70% are provided. (TIF) [file pgen.1006602.s001.tif]

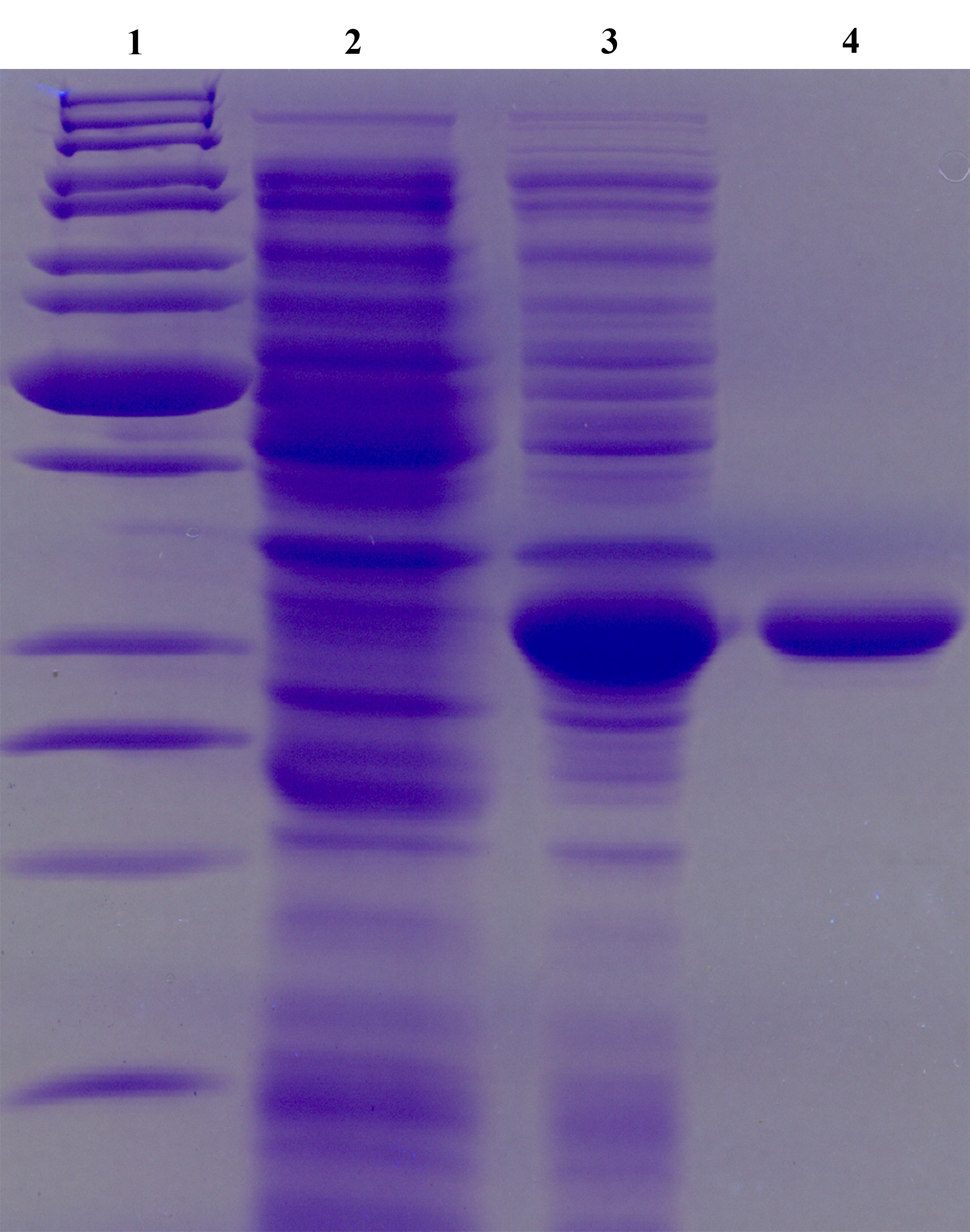

Supplement: S2 Fig — lane 1, molecular weight markers (10 kDa–200 kDa); lane 2, E. coli BL21 (DE3) harboring null vector pET28a; lane 3, extract of E. coli BL21 (DE3) harboring pET28a-ant; 4, Purified ANT(3")-IIa protein. (TIF) [file pgen.1006602.s002.tif]

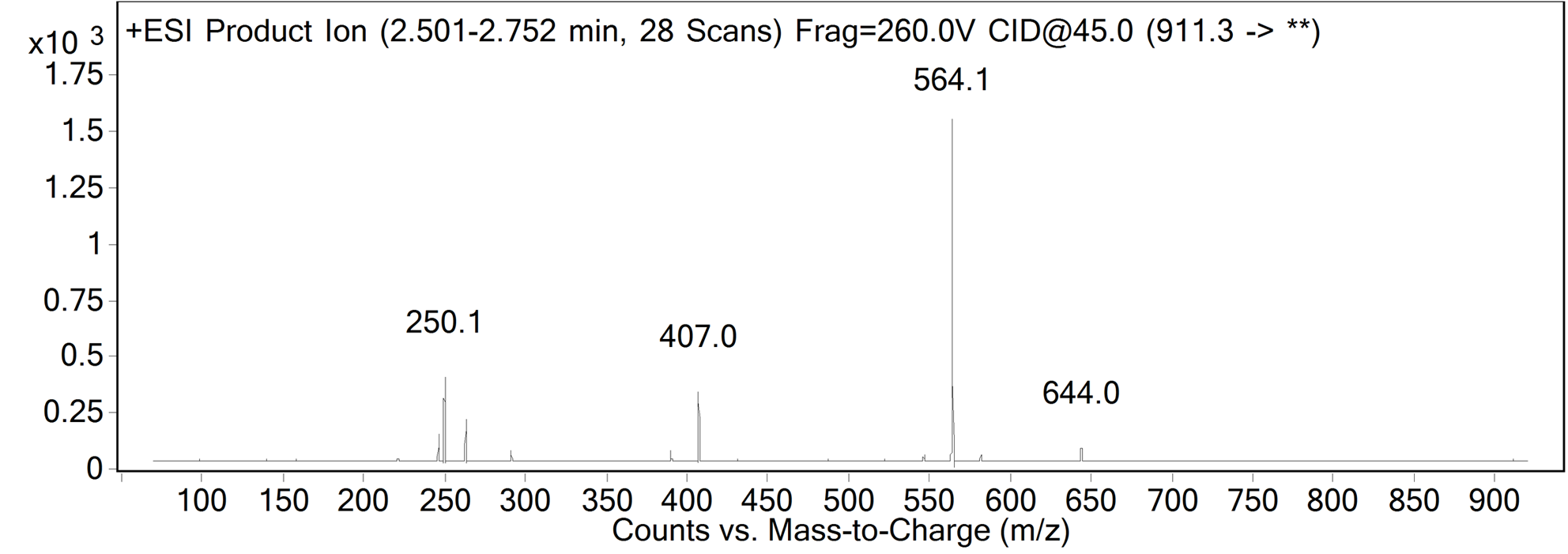

Supplement: S3 Fig — (TIF) [file pgen.1006602.s003.tif]

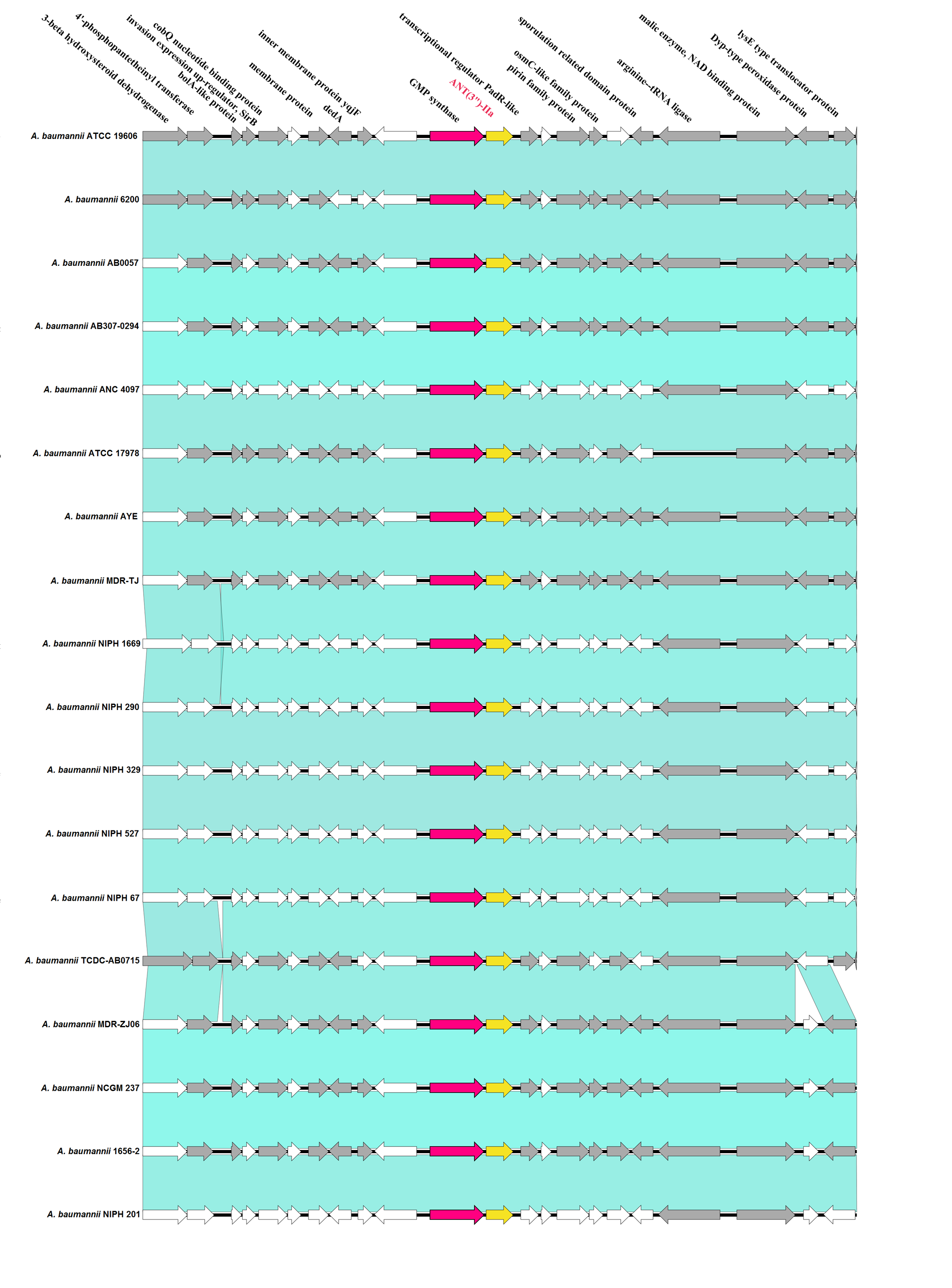

Supplement: S4 Fig — Aqua green indicates high nucleotide similarity (>98%). Arrows indicate open reading frames and the direction of transcription. Yellow arrow, ant(3")-IIa; Red arrow, GMP synthase gene. Boldfaced text above the arrows indicates the protein product of each gene. The displayed sequences of A. baumannii ATCC 19606, 6200, AB0057, AB307-0294, ANC 4097, 17978, AYE, MDR-TJ, NIPH 1669, NIPH 290, NIPH 329, NIPH 527, NIPH 67, TCDC-AB0715, MDR-ZJ06, NCGM 237, 1656–2 and NIPH 201 are subregions of Genbank accessions, JMRY01000011, NZ_CP010397, NC_011586, NC_011595, APRF01000002, CP000521, NC_010410, NC_017847, APOQ01000016, APRD01000022, APQY01000009, APQW01000014, APRA01000009, CP002522, NC_017171, NZ_AP013357, NC_017162 and APQV01000013, respectively. (TIF) [file pgen.1006602.s004.tif]

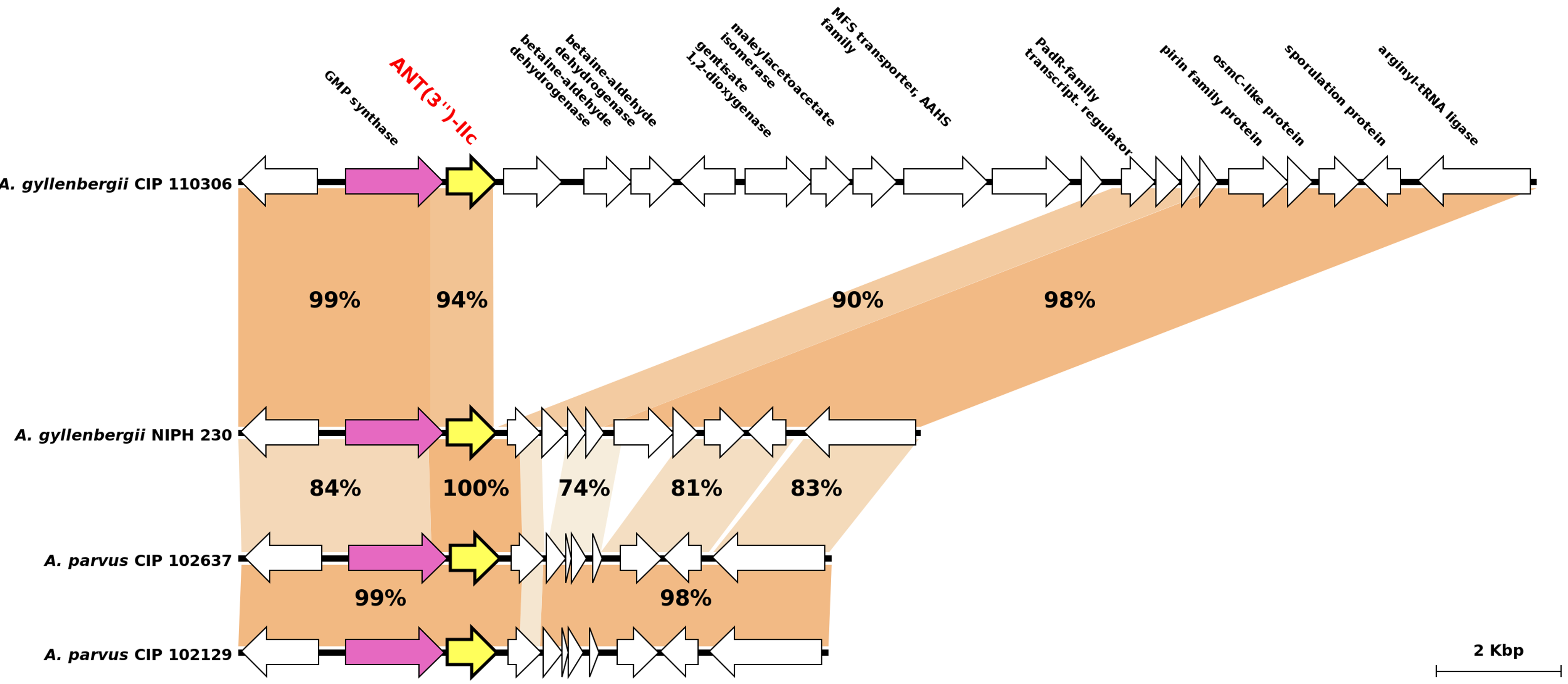

Supplement: S5 Fig — Deep orange indicates high nucleotide similarity, and light orange indicates low nucleotide similarity. Arrows indicate open reading frames and the direction of transcription. Boldfaced text above the arrows indicates the protein product of each gene. Yellow arrow, ant(3")-IIc; pink arrow, GMP synthase gene; genes without label, hypothetical proteins. The displayed sequences of A. gyllenbergii CIP 110306, A. gyllenbergii NIPH 230, A. parvus CIP 102637, and A. parvus CIP 102129 are subregions of Genbank accessions ATGG01000028, AYEQ01000163, APPG01000010, and APPA01000001, respectively. (TIF) [file pgen.1006602.s005.tif]

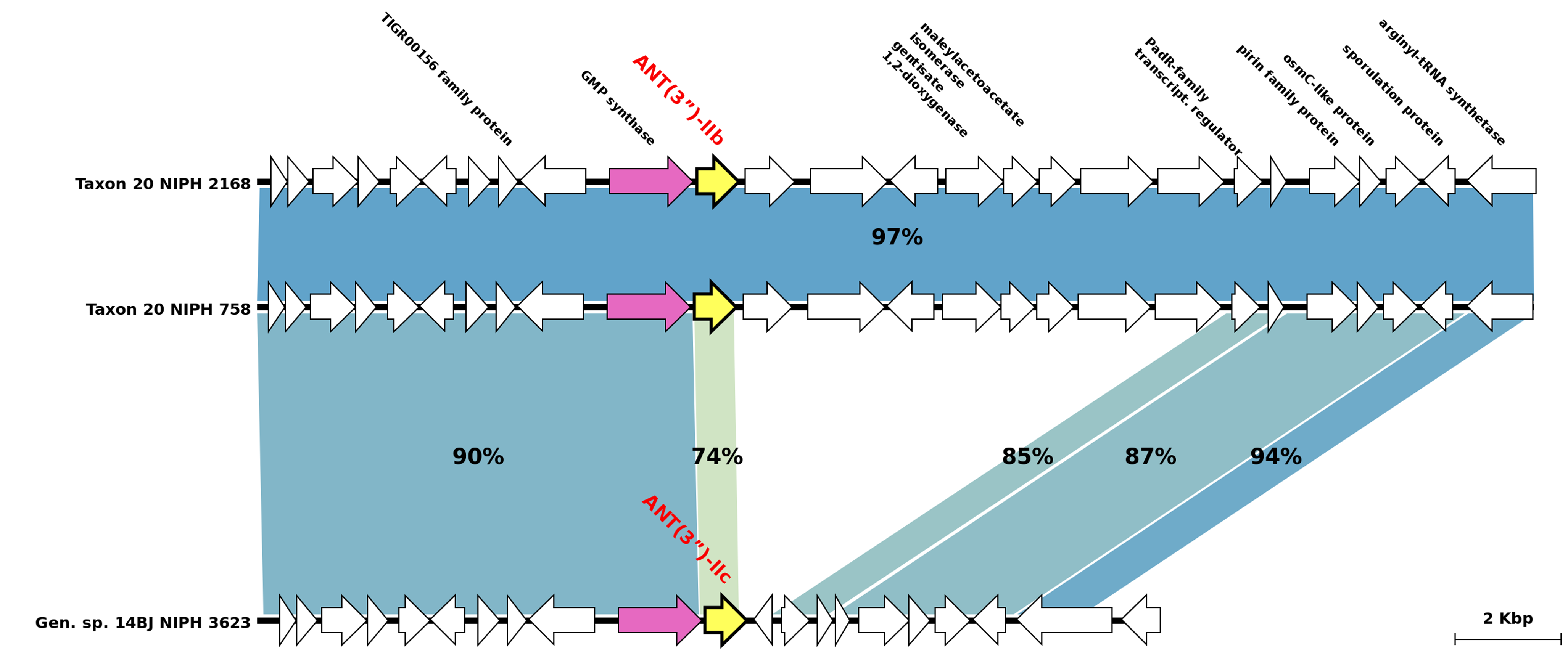

Supplement: S6 Fig — Blue indicates high nucleotide similarity, and light green indicates low nucleotide similarity. Arrows indicate open reading frames and the direction of transcription. Yellow arrow, ant(3")-II genes; Pink arrow, GMP synthase gene; genes without label, hypothetical proteins. The putative HR event encompassed ant(3")-IIb and the downstream inserted region in Taxon 20 NIPH 2168 and NIPH 758, leading to a low similarity region inside an otherwise more highly similar region, when compared to Gen. sp. 14BJ NIPH 3623. Sequences of A. sp. neg1 and B-65365 are essentially identical to sequences of Taxon 20 NIPH 2168 and NIPH 758 and are not represented here. The displayed sequences of Taxon 20 NIPH 2168, NIPH 758, and Gen. sp. 14BJ NIPH 3623 are subregions of Genbank accessions APRW01000008, APPC01000022, and APSA01000001, respectively. (TIF) [file pgen.1006602.s006.tif]

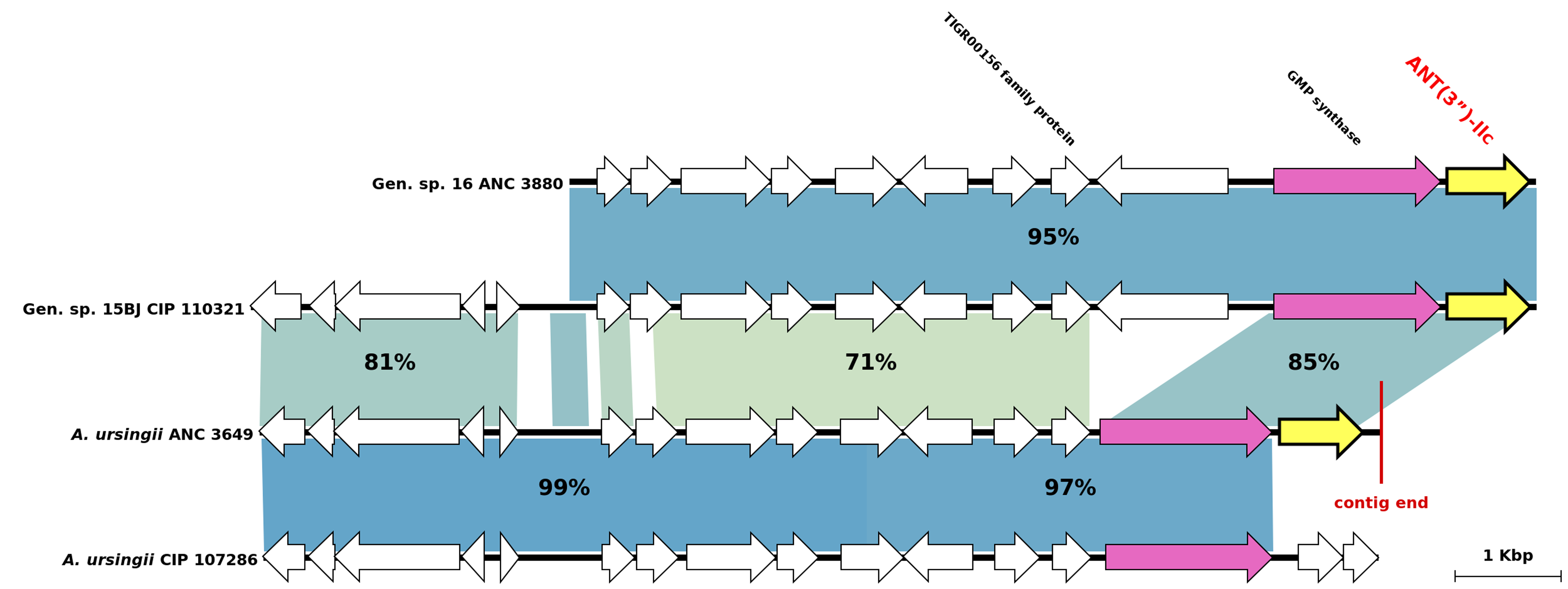

Supplement: S7 Fig — Blue indicates high nucleotide similarity, and light green indicates low nucleotide similarity. Arrows indicate open reading frames and the direction of transcription. Yellow arrow, ant(3")-IIc; Pink arrow, GMP synthetase gene; genes without label, hypothetical proteins. The putative HR region encompasses the ant(3")-IIc gene and the GMP synthase-encoding gene in A. ursignii ANC 3649. The displayed sequences of A. ursingii CIP 107286, ANC 3649, Gen. sp. 15BJ CIP 110321, and Gen. sp. 16 ANC 3880 are subregions of Genbank accessions APQA01000027, APQC01000003, AQFL01000027, and APSD01000005, respectively. (TIF) [file pgen.1006602.s007.tif]

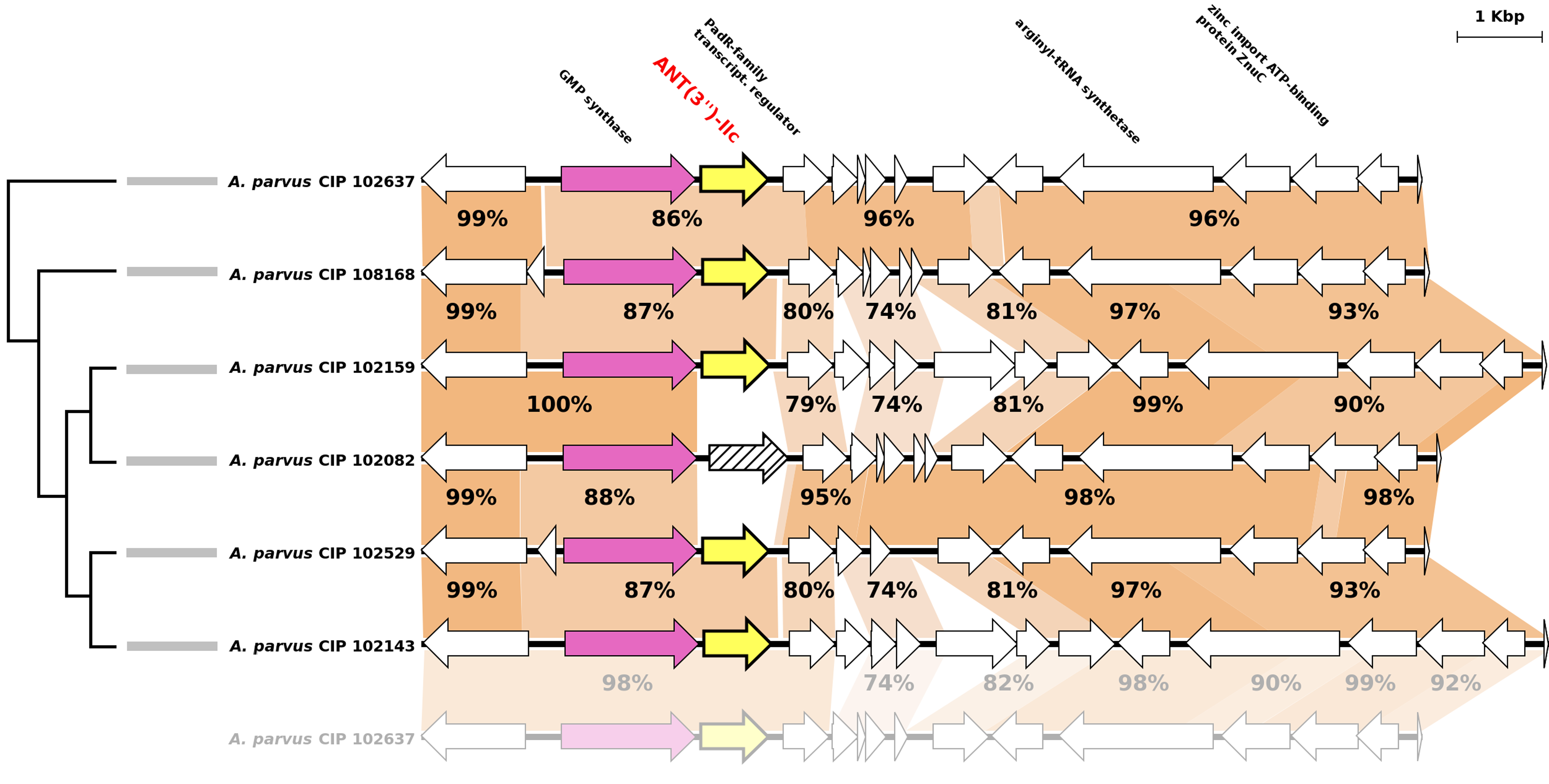

Supplement: S8 Fig — The phylogenetic relationship between strains as reported in Fig 2 is shown on the left side. Deep Orange indicates high nucleotide similarity, and light orange indicates low nucleotide similarity. Arrows indicate open reading frames and the direction of transcription. Yellow arrow, ant(3")-IIc; pink arrow, GMP synthase gene; striped arrow, restriction-modification gene; genes without label, hypothetical proteins. A. parvus CIP 102637 is repeated at the bottom of the figure to show its similarity with A. parvus CIP 102143 at the ant(3")-IIc locus. The nucleotide pairwise similarity at the ant(3")-IIc locus and the directly adjacent genes (including the GMP synthase and the PadR-family transcriptional regulator) is consistently lower than at the flanking regions, indicating multiple events of allele exchange through homologous recombination. ant(3")-IIc have also been precisely replaced by an unrelated gene in A. parvus CIP 102082. The displayed sequences of A. sp. A. parvus CIP 102637, CIP 108168, CIP 102159, CIP 102082, CIP 102529, and CIP 102143 are subregions of Genbank accessions APPG01000010, APOM01000001, KB851223, KB849372, KB849355, and APSE01000019, respectively. (TIF) [file pgen.1006602.s008.tif]
