# Supplementary material for: A new subclass of intrinsic aminoglycoside nucleotidyltransferases, ANT(3")-II, is horizontally transferred among Acinetobacter spp. by homologous recombination
Source: PLoS Genet. 2017 Feb 2;13(2):e1006602. doi: 10.1371/journal.pgen.1006602 (PMC5313234; doi:10.1371/journal.pgen.1006602)
Supplement: S1 Table — (DOCX) [file pgen.1006602.s009.docx]

S1 Table. Putative transferase resistance proteins located on *Acinetobacter* chromosomes

| Strains | Putative resistance proteins | Coverage  /Identity (%) | Closest known transferase |
| --- | --- | --- | --- |
| *A. baumannii* ATCC 19606 | EEX02086 | 84/40 | ANT(3")-Ia |
| *A. parvus* CIP 108168 | ENU37733 | 87/42 | ANT(3")-Ia |
| *A. gyllenbergii* NIPH 230 | ESK39014 | 85/44 | ANT(3")-Ia |
| Gen. sp. 13BJ/14TU NIPH 1859 | ENX32354 | 84/39 | ANT(3")-Ia |
| Taxon 20 NIPH 758 | ENU91137 | 84/41 | ANT(3")-Ia |
