# Supplementary material for: A new subclass of intrinsic aminoglycoside nucleotidyltransferases, ANT(3")-II, is horizontally transferred among Acinetobacter spp. by homologous recombination
Source: PLoS Genet. 2017 Feb 2;13(2):e1006602. doi: 10.1371/journal.pgen.1006602 (PMC5313234; doi:10.1371/journal.pgen.1006602)
Supplement: S2 Table — (DOCX) [file pgen.1006602.s010.docx]

S2 Table. Computational analysis of different HR algorithms with RDP4

| Methods | Av. P-val |
| --- | --- |
| RDP | 1.225×10^-257^ |
| GENECONV | 8.335×10^-240^ |
| BootScan | 2.097×10^-249^ |
| MaxChi | 6.692×10^-49^ |
| Chimaera | 6.160×10^-48^ |
| SiScan | 1.267×10^-44^ |
| PhylPro | 1.939×10^-224^ |
