# Supplementary material for: A new subclass of intrinsic aminoglycoside nucleotidyltransferases, ANT(3")-II, is horizontally transferred among Acinetobacter spp. by homologous recombination
Source: PLoS Genet. 2017 Feb 2;13(2):e1006602. doi: 10.1371/journal.pgen.1006602 (PMC5313234; doi:10.1371/journal.pgen.1006602)
Supplement: S3 Table — (DOCX) [file pgen.1006602.s011.docx]

S3 Table. Primers used to amplify the putative *ant(3")-IIa* and to construct gene-deficient mutant and complementation plasmid

| Primer | Sequence (5’–3’) | Inserted site |
| --- | --- | --- |
| antF | gatgaattcgatgcctgatttcattcagtt | *Eco*RI |
| antR | gataagcttgtcggtaaaaaagccagaat | *Hin*dIII |
| antUF | gatgaattcagcctttacgtgacttgttt | *Eco*RI |
| antUR | tcaaatttcttttctgctcaaaactgaatgaaatcaggcat |  |
| antDF | atgcctgatttcattcagttattgagcagaaaagaaatttga |  |
| antDR | gataagcttatgaggttcatttcttccag | *Hin*dIII |
| antTestF | taaatctcaccacaatgtgg |  |
| antTestR | ttagcttgtcttggtgttca |  |
