# Supplementary material for: A new subclass of intrinsic aminoglycoside nucleotidyltransferases, ANT(3")-II, is horizontally transferred among Acinetobacter spp. by homologous recombination
Source: PLoS Genet. 2017 Feb 2;13(2):e1006602. doi: 10.1371/journal.pgen.1006602 (PMC5313234; doi:10.1371/journal.pgen.1006602)
Supplement: S4 Table — (DOCX) [file pgen.1006602.s012.docx]

S4 Table. Sequence accession or GI number used in Fig 1.

| Strain | Enzyme | Accession/GI |
| --- | --- | --- |
| *Staphylococcus aureus* Tn554 | ANT(9)-Ia | CAA26428 |
| *Staphylococcus aureus* BK16691 (Tn6072) | ANT(9)-Ia | ADC53383 |
| *Enterococcus faecalis* LDR55 | ANT(9)-Ib | AAA16527 |
| *Klebsiella pneumoniae* NK 29 | ANT(3'')-I(*aadA*2) | YP_001965793 |
| *Yersinia pestis* biovar Orientalis str. IP275 | ANT(3'')-I | ABO42050 |
| *Escherichia coli* Plasmid NR79 | ANT(3'')-I(*aadA*3) | AAC14728 |
| Uncultured bacterium gca-Q | ANT(3'')-I(*aadA*8) | AAN41439 |
| *Pseudomonas aeruginosa* C1-34 | ANT(3'')-I(*aadA*15) | ABD58917 |
| *Aeromonas media* ER.1.18 | ANT(3'')-I(*aadA*17) | ACK43806 |
| *Escherichia coli* taxon 562 | ANT(3'')-I(*aadA*12) | ACJ47200 |
| *Escherichia coli* plasmid R538-1 | ANT(3'')-Ia(*aadA*1) | CAA26199 |
| *Salmonella enterica* subsp. enterica serovar Newport 9854 | ANT(3'')-I(*aadA*24) | ABG72894 |
| *Salmonella enterica* subsp. enterica serovar Typhimurium CNM 179-02 | ANT(3'')-I(*aadA*22) | CAK12750 |
| *Salmonella enterica* subsp. enterica serovar Agona 231 | ANT(3'')-I(*aadA*23) | CAH10847 |
| *Salmonella enterica* subsp. enterica serovar Typhimurium ACSSuT | ANT(3'')-I(*aadA*21) | AAN87151 |
| *Escherichia coli* CCUG 47655 | ANT(3'')-I(*aadA*11) | CAD99015 |
| *Providencia rettgeri* R7K | ANT(3'')-I(*aadA*13) | YP_001874900 |
| *Vibrio fluvialis* H-08942 | ANT(3'')-I(*aadA*7) | BAD00739 |
| *Escherichia coli* 1387 | ANT(3'')-I(*aadA*16) | ACF17980 |
| *Pseudomonas aeruginosa* NIPH 804-03 | ANT(3'')-I(*aadA*10) | CAJ32491 |
| *Pseudomonas aeruginosa* NIPH 2611 | ANT(3)-I(*aadA*6) | CAJ32504 |
| *Pasteurella multocida* 6647 | ANT(3'')-I(*aadA*14) | CAI57696 |
| *Escherichia coli* 9516014-1 | ANT(3'')-I(*aadA*5) | AAF17880 |
| *Escherichia coli* pIP1206 | ANT(3'')-I(*aadA*4) | YP_001816600 |
| *Corynebacterium glutamicum* LP-6 | ANT(3'')-I(*aadA*9) | NP_478099.1 |
| *Acinetobacter baumannii* AP | ANT(3'')-I (*aadA*2) | ADU56648 |
| *Acinetobacter pittii* YMCU160 | ANT(3'')-I (*aadA*1) | AAK54202 |
| *Acinetobacter baumannii* 26 | ANT(3'')-I (*aadA*15) | ADW09008 |
| *Acinetobacter junii* M-B10A | ANT(3'')-I (*aadA*1) | AIA09246 |
| *Pasteurella multocida* 36950 | ANT(3'')-I (*aadA*25) | AET15272 |
| *Acinetobacter baumannii* NIPH 290 | ANT(3'')-IIa | ENW60207 |
| *Acinetobacter baumannii* NIPH 527 | ANT(3'')-IIa | ENW33737 |
| *Acinetobacter baumannii* MRSN 3405 | ANT(3'')-IIa | KLT93807 |
| *Acinetobacter baumannii* AYE | ANT(3'')-IIa | CAJ77832 |
| *Acinetobacter baumannii* ANC 4097 | ANT(3'')-IIa | ENW71917 |
| *Acinetobacter baumannii* AB307-0294 | ANT(3'')-IIa | ACJ58575 |
| *Acinetobacter baumannii* AB0057 | ANT(3'')-IIa | ACJ39602 |
| *Acinetobacter baumannii* NIPH 615 | ANT(3'')-IIa | ENU71562 |
| *Acinetobacter baumannii* TCDC-AB0715 | ANT(3'')-IIa | ADX90594 |
| *Acinetobacter baumannii* NIPH 335 | ANT(3'')-IIa | ENW40943 |
| *Acinetobacter baumannii* NIPH 80 | ANT(3'')-IIa | ENW75567 |
| *Acinetobacter baumannii* NIPH 410 | ANT(3'')-IIa | EPG34384 |
| *Acinetobacter baumannii* NIPH 70 | ANT(3'')-IIa | ENW58248 |
| *Acinetobacter baumannii* SDF | ANT(3'')-IIa | CAJ31119 |
| *Acinetobacter baumannii* ATCC 17978 | ANT(3'')-IIa | ABO10616 |
| *Acinetobacter baumannii* NIPH 1362 | ANT(3'')-IIa | ENU53612 |
| *Acinetobacter baumannii* MDR-TJ | ANT(3'')-IIa | AFI97223 |
| *Acinetobacter baumannii* NIPH 329 | ANT(3'')-IIa | ENW42167 |
| *Acinetobacter baumannii* NIPH 1669 | ANT(3'')-IIa | ENU49542 |
| *Acinetobacter baumannii* NIPH 190 | ANT(3'')-IIa | ENV24220 |
| *Acinetobacter baumannii* ATCC 19606 | ANT(3'')-IIa | EEX02086 |
| *Acinetobacter baumannii* NIPH 201 | ANT(3'')-IIa | ENW33126 |
| *Acinetobacter baumannii* 1656-2 | ANT(3'')-IIa | ADX01798 |
| *Acinetobacter baumannii* MDR-ZJ06 | ANT(3'')-IIa | AEP04618 |
| *Acinetobacter baumannii* NIPH 24 | ANT(3'')-IIa | ENU11423 |
| *Acinetobacter baumannii* NIPH 528 | ANT(3'')-IIa | ENW59610 |
| *Acinetobacter baumannii* NIPH 1734 | ANT(3'')-IIa | ENU75549 |
| *Acinetobacter baumannii* NIPH 2061 | ANT(3'')-IIa | ENU75336 |
| *Acinetobacter baumannii* NIPH 67 | ANT(3'')-IIa | ENW48320 |
| *Acinetobacter baumannii* NIPH 146 | ANT(3'')-IIa | ENU67239 |
| *Acinetobacter baumannii* NIPH 601 | ANT(3'')-IIa | ENW49716 |
| *Acinetobacter junii* NBRC 109759 | ANT(3'')-IIa | GI750115791:c9085-8300 |
| *Acinetobacter junii* TG19608 | ANT(3'')-IIa | AMJF01000087:c9139-8354 |
| *Acinetobacter junii* CIP 64.5 | ANT(3'')-IIa | ENV65326 |
| *Acinetobacter pittii* NBRC 110508 | ANT(3'')-IIa | GI757728111:64521-65240 |
| *Acinetobacter pittii* SH024 | ANT(3'')-IIa | ADCH01000058:c35613-34894 |
| *Acinetobacter pittii* ABBL047 | ANT(3'')-IIa | KRJ76323 |
| *Acinetobacter pittii* ABBL010 | ANT(3'')-IIa | KRI28448 |
| *Acinetobacter pittii* ABBL065 | ANT(3'')-IIa | KQD30668 |
| *Acinetobacter pittii* ABBL033 | ANT(3'')-IIa | KRJ16515 |
| *Acinetobacter pittii* ABBL074 | ANT(3'')-IIa | KQE06243 |
| *Acinetobacter pittii* ABBL096 | ANT(3'')-IIa | KQE84386 |
| *Acinetobacter pittii* ABBL120 | ANT(3'')-IIa | KQF50070 |
| *Acinetobacter pittii* ABBL103 | ANT(3'')-IIa | KQF02724 |
| *Acinetobacter* *pittii* PHEA-2 | ANT(3'')-IIa | ADY83642 |
| *Acinetobacter pittii* ABBL148 | ANT(3'')-IIa | KQG39930 |
| *Acinetobacter pittii* ABBL078 | ANT(3'')-IIa | KQE30712 |
| *Acinetobacter pittii* ABBL005 | ANT(3'')-IIa | KRI14539 |
| *Acinetobacter pittii* ABBL019 | ANT(3'')-IIa | KRI64217 |
| *Acinetobacter pittii* DSM 25618 | ANT(3'')-IIa | GI752490455:c35450-34665 |
| *Acinetobacter pittii* DSM 21653 | ANT(3'')-IIa | AIEK01000003:58850-59635 |
| *Acinetobacter pittii* ABBL024 | ANT(3'')-IIa | KRI81017 |
| *Acinetobacter pittii* ABBL046 | ANT(3'')-IIa | KRJ63537 |
| *Acinetobacter pittii* ABBL077 | ANT(3'')-IIa | KQE23279 |
| *Acinetobacter pittii* ABBL086 | ANT(3'')-IIa | KQE42912 |
| *Acinetobacter pittii* ABBL126 | ANT(3'')-IIa | KQF75196 |
| *Acinetobacter pittii* ABBL064 | ANT(3'')-IIa | KQD39257 |
| *Acinetobacter pittii* ANC 3678 | ANT(3'')-IIa | ENW09672 |
| *Acinetobacter pittii* ABBL128 | ANT(3'')-IIa | KQG06409 |
| *Acinetobacter pittii* CIP 70.29 | ANT(3'')-IIa | ENW14756 |
| *Acinetobacter pittii* ABBL015 | ANT(3'')-IIa | KRI50457 |
| *Acinetobacter pittii* ABBL075 | ANT(3'')-IIa | KQE18073 |
| *Acinetobacter pittii* ABBL111 | ANT(3'')-IIa | KQF37071 |
| *Acinetobacter pittii* ABBL135 | ANT(3'')-IIa | KQG01287 |
| *Acinetobacter pittii* NBRC 110509 | ANT(3'')-IIa | GI758800041:59498-60217 |
| *Acinetobacter pittii* NBRC 110507 | ANT(3'')-IIa | GI 758799941:c35888-35169 |
| *Acinetobacter pittii* CR12-42 | ANT(3'')-IIa | JQNT01000039:25371-26090 |
| *Acinetobacter pittii* NBRC 110504 | ANT(3'')-IIa | GI757727632:c35888-35169 |
| *Acinetobacter* sp. neg1 | ANT(3'')-IIb | KHF75484 |
| Taxon 20 NIPH 2168 | ANT(3'')-IIb | ENX23950 |
| *Acinetobacter* sp. NRRL B-65365 | ANT(3'')-IIb | KYQ85042 |
| Taxon 20 NIPH 758 | ANT(3'')-IIb | ENU91137 |
| *Acinetobacter* sp. NCTC 7422 | ANT(3'')-IIc | AIED01000007:65654-66433 |
| *Acinetobacter* sp. NBRC 110496 | ANT(3'')-IIc | [BBTF01000017](https://www.ncbi.nlm.nih.gov/nuccore/BBTF01000017#_blank):11305-12084 |
| Gen. sp. 13BJ/14TU CIP 64.2 | ANT(3'')-IIc | ENX16268 |
| Gen. sp. 13BJ/14TU NIPH 2036 | ANT(3'')-IIc | EPG35341 |
| Gen. sp. 13BJ/14TU NIPH 1859 | ANT(3'')-IIc | ENX32354 |
| *Acinetobacter* *parvus* CIP 102129 | ANT(3'')-IIc | ENU87633 |
| *Acinetobacter* *parvus* CIP 102637 | ANT(3'')-IIc | ENV06939 |
| *Acinetobacter* *gyllenbergii* NIPH 230 | ANT(3'')-IIc | ESK39014 |
| *Acinetobacter* *parvus* CIP 102143 | ANT(3'')-IIc | ENX71736 |
| *Acinetobacter* *parvus* CIP 102159 | ANT(3'')-IIc | ENU83999 |
| *Acinetobacter* *gyllenbergii* MTCC 11365 | ANT(3'')-IIc | EPH31798 |
| *Acinetobacter* *gyllenbergii* GTC 14627 | ANT(3'')-IIc | GI757728617:c59333-58551 |
| Taxon 19 NIPH 809 | ANT(3'')-IIc | ENU22375 |
| *Acinetobacter* *gyllenbergii* CIP 110306 | ANT(3'')-IIc | EPF74767 |
| *Acinetobacter* *ursingii* ANC 3649 | ANT(3'')-IIc | ENV80847 |
| *Acinetobacter* sp. MN12 | ANT(3'')-IIc | WP_033133956 |
| Gen. sp. 16 CIP 56.2 | ANT(3'')-IIc | ENV10579 |
| Gen. sp. 16 CIP 70.18 | ANT(3'')-IIc | ENX61713 |
| *Acinetobacter* sp. TG19627 | ANT(3'')-IIc | [AMJM01000099](https://www.ncbi.nlm.nih.gov/nuccore/AMJM01000099#_blank):53327-54103 |
| Gen. sp. 16 ANC 3880 | ANT(3'')-IIc | ENX64487 |
| Gen. sp. 15BJ CIP 110321 | ANT(3'')-IIc | EOR03352 |
| Gen. sp. 14BJ. NIPH 3623 | ANT(3'')-IIc | ENX40876 |
| *Acinetobacter* sp. WC-323 | ANT(3'')-IIc | EKU57184 |
| *Acinetobacter* *parvus* GTC 3704 | ANT(3'')-IIc | GI960501059:c35703-34924 |
| Taxon 18-like NIPH 284 | ANT(3'')-IIc | ENW85432 |
| *Acinetobacter* *parvus* CIP 102529 | ANT(3'')-IIc | ENU87960 |
| *Acinetobacter* *parvus* CIP 108168 | ANT(3'')-IIc | ENU37733 |
